# Claudin1 decrease induced by 1,25-dihydroxy-vitamin D3 potentiates gefitinib resistance therapy through inhibiting AKT activation-mediated cancer stem-like properties in NSCLC cells

**DOI:** 10.1038/s41420-022-00918-5

**Published:** 2022-03-18

**Authors:** Zhirong Jia, Kaiwei Wang, Yalei Duan, Kaiyong Hu, Yameng Zhang, Meisa Wang, Kang Xiao, Shuo Liu, Zhenzhen Pan, Xuansheng Ding

**Affiliations:** 1 School of Basic Medicine and Clinical Pharmacy, China Pharmaceutical University, 211198 Nanjing, China; 2 R&D Department, Hubei Monyan Pharmaceutical Co., Ltd, 448124 Jingmen, China; 3 Precision Medicine Laboratory, School of Basic Medicine and Clinical Pharmacy, China Pharmaceutical University, 211198 Nanjing, China; 4 Medical School, Anhui University of Science and Technology, 232001 Huainan, China

**Keywords:** Lung cancer, Cancer

## Abstract

Claudins, the integral tight junction proteins that regulate paracellular permeability and cell polarity, are frequently dysregulated in cancer; however, their roles in regulating EGFR tyrosine kinase inhibitors (EGFR-TKIs) resistance in non-small cell lung cancer (NSCLC) are unknown. To this end, we performed GEO dataset analysis and identified that claudin1 was a critical regulator of EGFR-TKI resistance in NSCLC cells. We also found that claudin1, which was highly induced by continuous gefitinib treatment, was significantly upregulated in EGFR-TKI-resistant NSCLC cells. By knocking down claudin1 in cell lines and xenograft models, we established that gefitinib resistance was decreased. Moreover, claudin1 knockdown suppressed the expression levels of pluripotency markers (Oct4, Nanog, Sox2, CD133, and ALDH1A1). Claudin1 loss inhibited phosphorylated AKT (p-AKT) expression and reduced cancer cell stemness by suppressing AKT activation. Furthermore, SKL2001, a β-catenin agonist, upregulated the expression levels of claudin1, p-AKT, and pluripotency markers, and 1,25-dihydroxy-vitamin D3 (1,25(OH)_2_D_3_) reduced claudin1 expression, AKT activation, and cancer cell stemness by inhibiting β-catenin, and suppressed claudin1/AKT pathway mediated cancer stem-like properties and gefitinib resistance. Collectively, inhibition of claudin1-mediated cancer stem-like properties by 1,25(OH)_2_D_3_ may decrease gefitinib resistance through the AKT pathway, which may be a promising therapeutic strategy for inhibiting gefitinib resistance in EGFR-mutant lung adenocarcinoma.

## Introduction

Lung cancer has been noted due to the increasing rate of morbidity and mortality worldwide [1]. Many patients with lung adenocarcinoma (LUAD), a major subtype of lung cancer [2], harbor mutations in the epidermal growth factor receptor (EGFR) in their cancer tissues and initially react well to molecular targeted drugs such as gefitinib, which inhibits EGFR tyrosine kinase (TKI) [3, 4]. However, acquired drug resistance inevitably occurs within 10–14 months, leading to poor prognostic outcomes [5, 6]. Some of this variability is associated with pre-existing EGFR T790M mutations that are resistant to first-generation TKIs. However, even though there are newer generation drugs that are highly effective against this subclone (such as osimertinib), a subpopulation of cells survive, enabling the development of other resistance mechanisms [7]. Therefore, there is a critical need to identify the mechanisms and potential novel therapeutic targets for EGFR-TKI resistance in order to develop strategies for overcoming EGFR-TKI resistance.

Claudins are major integral membrane tight junction proteins that are vital in the regulation of defense and barrier functions, as well as differentiation and polarity in epithelial and endothelial cells. Altered expression levels of several claudin proteins, in particular claudin1, 3, 4, and 7 have been associated with the development of various cancers, such as those involved in cancer cell proliferation, growth, survival, migration, invasion, and metastasis [8]. Claudins play critical roles in formation of cancer stem cells or tumor-initiating cells (CSCs/TICs) [9]. Notably, the acquisition of cancer stem-like properties contributes to EGFR-TKI resistance in NSCLC cells. Gefitinib/osimertinib-resistant NSCLC cells and clinical samples with acquired resistance to EGFR-TKIs exhibit elevated expression of stem cell-related markers, including ALDH1A1, Sox2, Oct4, and Nanog, and acquire stem cell-like properties [10–12]. In addition, the EGFR-TKI-induced phenotype of stem cell-like cancer cells enhances the invasion and migration abilities of drug-resistant cells [13]. Despite the critical role of claudin proteins in regulating the invasive and CSC-like properties of cancer cells, the effects and mechanisms of claudin proteins in the regulation of EGFR-TKI resistance are unknown. Elucidation of the emerging role of claudins in cancer stem-like properties and EGFR-TKI resistance as well as how to regulate claudins expression may inform the development of effective therapies against EGFR-TKI resistance.

Emerging evidence support the idea that 1,25-dihydroxyvitamin D3 (1,25(OH)_2_D_3_, 1,25D, calcitriol) inhibits lung cancer cell proliferation [14, 15] and opposes erlotinib or osimertinib resistance in preclinical models or in EGFR-mutant LUAD patients [16, 17]. Notably, 1,25D could restrain cancer cell stemness, invasion, and metastasis in various cancer cells [18–21]. Our recent research demonstrates that 1,25(OH)_2_D_3_ inhibits cancer stem-like properties and reverses gefitinib resistance in PC9/GR cells [22]. Therefore, 1,25(OH)_2_D_3_ may play an important role in the regulation of EGFR-TKI resistance in NSCLC cells and the mechanism may be closely associated with its regulation of cancer stem-like properties. Given that both 1,25(OH)_2_D_3_ and claudin proteins regulate cancer cell stemness, we postulate that there is an association between 1,25(OH)_2_D_3_ and claudins.

In this study, for the first time, we identify that the claudin proteins play an important role in EGFR-TKI resistance and determine the effects and mechanisms of this claudin protein in the regulation of gefitinib resistance and cancer stem-like properties. Moreover, we also examine whether and how 1,25(OH)_2_D_3_ causally regulates the expression and function of this claudin protein in EGFR-mutated NSCLC.

## Results

### Claudin1 is positively correlated with EGFR-TKI resistance

**Fig. 1:**
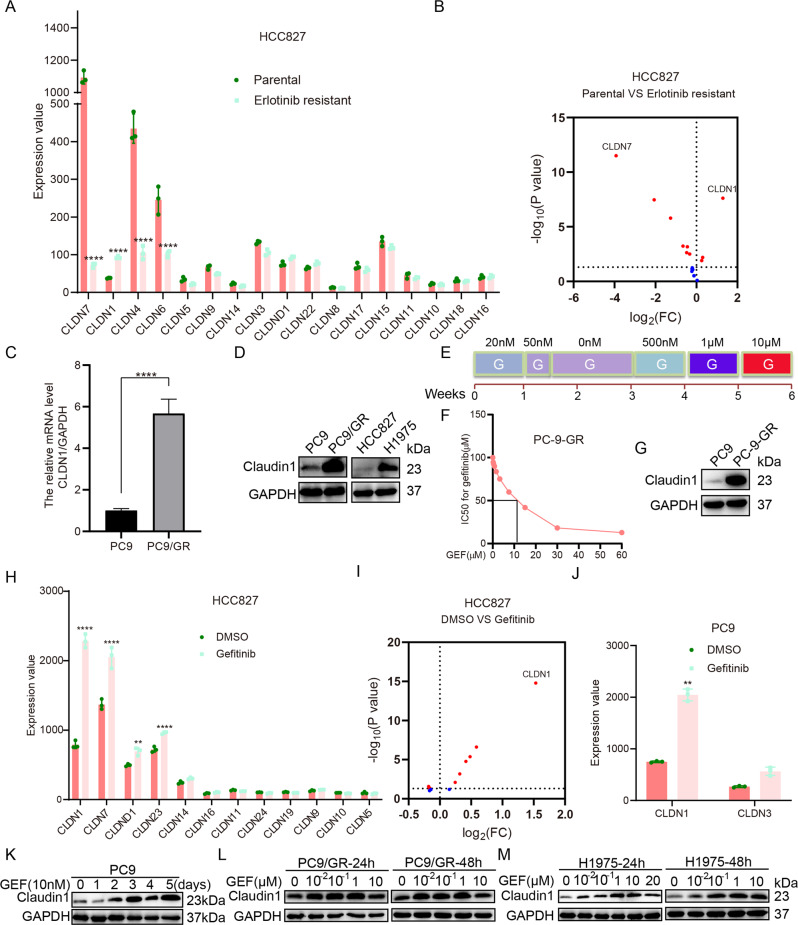
Claudin1 is positively correlated with EGFR-TKI resistance. **A**, **B** Significantly downregulated and upregulated mRNAs in erlotinib-sensitive and erlotinib-resistant HCC827 cells were analyzed. Statistical comparisons were performed using a one-way analysis of variance with Dunnett’s test. *n* = 3, ****P < 0.0001. Volcano map: Upregulated: *P* < 0.05, log_2_FC > 0; Downregulated: *P* < 0.05, log_2_FC < 0. **C** Relative mRNA expression levels of *CLDN1* in PC9 and PC9/GR cells were detected by real-time PCR. ****P < 0.0001. **D** Western blotting was performed to determine claudin1 expression in PC9, PC9/GR, HCC827, and H1975 cells. **E** Induction of gefitinib resistance in PC9 cells: PC9 cells were first treated with gefitinib at a concentration of 20 nM for 1 week. A small number of remaining cells were treated for another 2 days with a concentration of 50 nM, which was sufficient to kill nearly all parental cells. The remaining few cells were continuously cultured in the absence of gefitinib for 2 weeks. Then, cells were sequentially treated with gefitinib at concentrations of 500 nM for 1 week, 1 μM for another 1 week and 10 μM for the last 1 week. **F**, **G** Cell viability was determined by CCK-8 and claudin1 expression was detected by western blotting. **H**, **I** Significantly downregulated and upregulated *CLDNs* in gefitinib treated or untreated HCC827 cells. Statistical comparisons were performed using a one-way analysis of variance with Dunnett’s test. *n* = 3, **P < 0.01, ****P < 0.0001. Volcano map: Upregulated: *P* < 0.05, log_2_FC > 0; Downregulated: *P* < 0.05, log_2_FC < 0. **J** Significantly altered *CLDNs* mRNA expression levels in gefitinib-treated or untreated PC9 cells were evaluated. *n* = 3, **P < 0.01. **K** PC9 cells were treated with gefitinib (10 nM) after which claudin1 expression levels at the indicated time points were determined. **L**, **M** PC9/GR and H1975 cells were treated with the indicated concentrations of gefitinib for 24 or 48 h. Claudin1 expression was assessed by western blotting.

According to the primary data from the NCBI GEO database, the expression levels of genes encoding claudin proteins in erlotinib-sensitive and erlotinib-resistant HCC827 cells were analyzed (Fig. 1A). We found that *CLDN1* was significantly upregulated in erlotinib-resistant HCC827 cells when compared to erlotinib-sensitive HCC827 cells (Fig. 1B). Moreover, compared to PC9 cells, transcript levels of *CLDN1* expression in PC9/GR cells were elevated significantly (Fig. 1C). Compared to gefitinib-sensitive PC9 and HCC827 cells, claudin1 expression in gefitinib-resistant PC9/GR and H1975 cells was significantly elevated (Fig. 1D and Supplemental Fig. S1A, B and SaA). In addition, when gefitinib resistance was induced in PC9 cells (Fig. 1E, F), claudin1 expression in PC-9-GR cells was upregulated, when compared to parental cells (Fig. 1G and Supplemental Fig. S1C, SaB). Then, our analysis using GEO datasets showed that after short-term induction of gefitinib resistance in HCC827 and PC9 cells, *CLDN1* expression was significantly upregulated among all *CLDNs* (Fig. 1H–J). Interestingly, after PC9 cells had been treated with 10 nM of gefitinib, claudin1 expression was gradually upregulated as exposure time to gefitinib was increased (Fig. 1K and Supplemental Fig. S1D and SaC). In PC9/GR cells, claudin1 expression was significantly upregulated by 0.01, 0.1, and 1 μM of gefitinib (Fig. 1L and Supplemental Fig. S1E, F and SaD). In H1975 cells, 0.01, 0.1, 1, 10, and 20 μM of gefitinib significantly elevated claudin1 levels (Fig. 1M, Supplemental Fig. S1G, H and SaE). These findings suggest that claudin1 expression is upregulated in EGFR-TKI resistant NSCLC cells.

### Claudin1 downregulation decreases gefitinib resistance in NSCLC cells

**Fig. 2:**
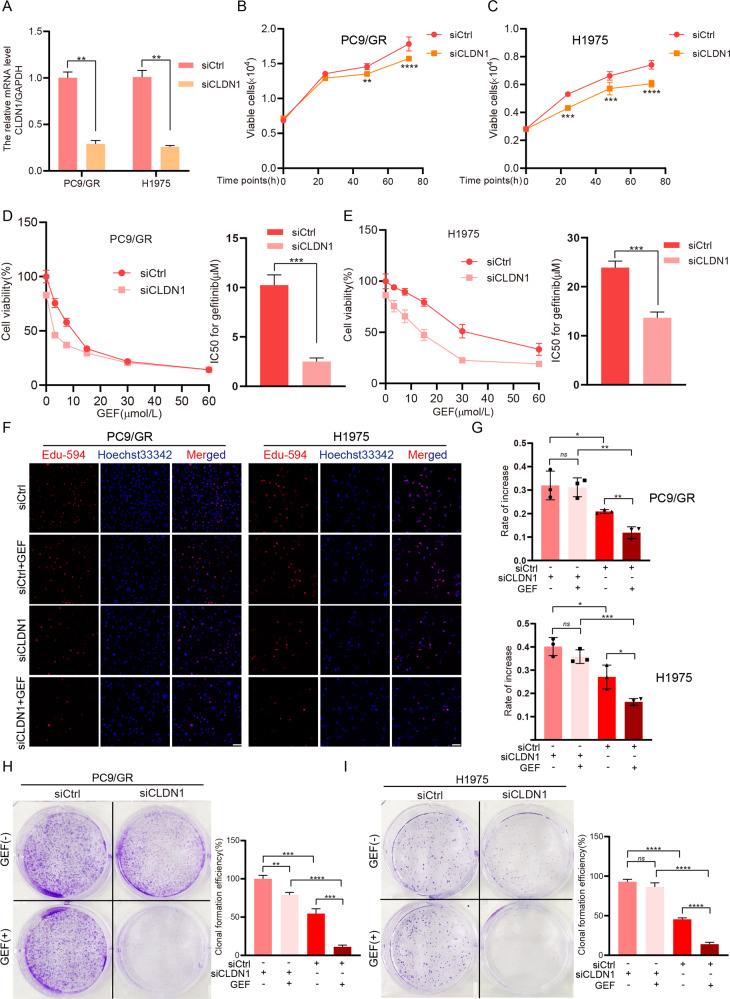
Claudin1 knockdown increases gefitinib sensitivity in NSCLC cells. **A** PC9/GR and H1975 cells were transfected with siRNA targeting CLDN1 for 24 h and relative mRNA levels were detected by real-time PCR. **P < 0.01. **B**, **C** PC9/GR and H1975 cells were transfected with siCtrl or siCLDN1, and viable cells at the indicated time points were counted. **P < 0.01, ***P < 0.001, ****P < 0.0001. **D**, **E** siRNA was transfected, after 6 h, various concentrations of gefitinib (0, 3.25, 7.5, 15, 30, 60 μmol/L) were, respectively, co-administered with siRNA for 24 and 48 h. Then the MTT assay was performed to determine cytotoxicity and the IC50 value against gefitinib was calculated (mean ± SD; *n* = 3; ****P < 0.0001). **F**, **G** PC9/GR and H1975 cells were transfected with siRNA, after 6 h, cells were treated with gefitinib (1 μM) for another 48 h and then stained for Edu (Scale bar: 100 μm; original magnification: ×100; representative images from three experiments). Cell proliferation rates were calculated as a percentage of Edu-positive nuclei to total nuclei (mean ± SD; *n* = 3; ns: not significant, *P < 0.05, **P < 0.01, ***P < 0.001). **H**, **I** PC9/GR and H1975 cells were transfected with siRNA, after 6 h, cells were treated with or without gefitinib (1 μM). Treatments were repeated every 3 days. Colony formation was assessed by crystal violet staining. Colony numbers were assessed by using the ImageJ software and clonal formation efficiency was calculated (**P < 0.01, ***P < 0.001, ****P < 0.0001).

Claudin1 knockdown significantly reduced cell viability (Fig. 2A–C) in PC9/GR and H1975 cells. The detection of cell viability (Fig. 2D, E), proliferation (Fig. 2F, G), and colony formation (Fig. 2H, I) indicated that the cells treated with the combination of knocking down claudin1 and gefitinib were more sensitive to gefitinib than that of the single treatment group. These findings imply that claudin1 is a promising therapeutic target for EGFR-TKI resistant NSCLC cells, underscoring the significance of claudin1 as a predictor of EGFR-TKI resistance.

### In vivo antitumor effects of combined claudin1 knockdown and gefitinib therapy

**Fig. 3:**
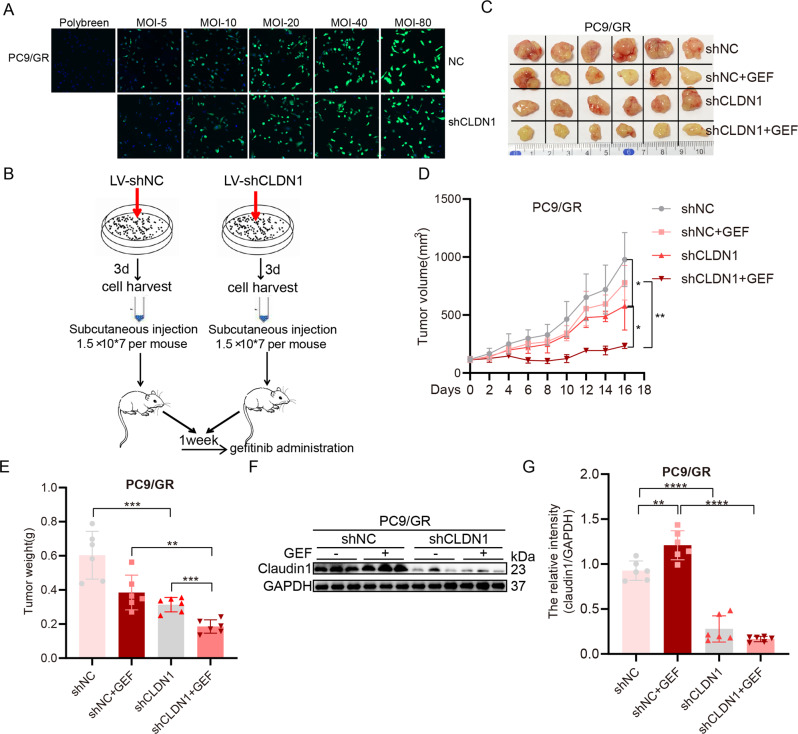
Targeting claudin1 suppresses lung cancer growth and resistance to gefitinib in mouse xenograft models. **A** PC9/GR cells were infected with a lentivirus for 72 h and infection efficiency was detected by a laser scanning confocal microscope. Note: Numbers represent MOI values; lentivirus concentrations: 3.0 × 10^8^ TU/mL. **B** Flow charts for the establishment of xenograft mice models. **C** Macroscopic appearance of xenografts in each group. **D**, **E** Tumor sizes and weight of PC9/GR xenograft models were presented as mean ± SD; *n* = 6; *P < 0.05, **P < 0.01, ***P < 0.001. **F** Whole protein cell lysates were randomly prepared from three tumors per group for western blotting to detect the indicated proteins. **G** Relative intensity of claudin1 protein was evaluated by the ChemiScope analysis software (mean ± SD; **P < 0.01, ****P < 0.0001).

**Fig. 4:**
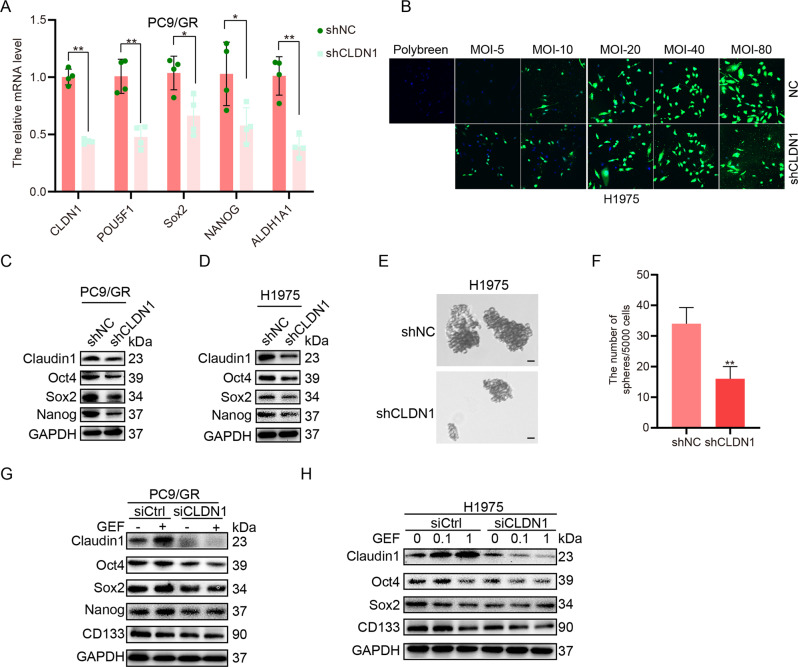
Claudin1 knockdown inhibits cancer cell stemness in NSCLC cells. **A** PC9/GR cells were infected with a lentivirus for 48 h. Then, total RNA were extracted and mRNA expression levels of *CLDN1*, *POU5F1*, *Sox2*, *NANOG*, and *ALDH1A1* were detected by real-time PCR (mean ± SD; *n* = 6; *P < 0.05, **P < 0.01). **B** H1975 cells were infected with a lentivirus for 72 h and infection efficiency was detected by a laser scanning confocal microscope. Note: Numbers represent MOI values; lentivirus concentration: 3.0 × 10^8^ TU/mL. **C**, **D** PC9/GR and H1975 cells were infected with lentivirus for 48 h and the expression of claudin1, Nanog, Sox2, and Oct4 was evaluated by western blotting. **E**, **F** Sphere formation of H1975 cells was observed after claudin1 knockdown. Cell spheres that were characterized by tight, spherical, non-adherent colonies of >90 μm in diameter were counted (Scale bar: 100 μm; original magnification: ×100; representative images from three experiments; mean ± SD; **P < 0.01). **G** siRNA was transfected for 6 h, then PC9/GR cells were treated with gefitinib (1 μM) for another 48 h, the protein was extracted and expression levels of claudin1, Oct4, Sox2, Nanog, and CD133 were evaluated by western blotting. **H** H1975 cells were transfected with siCLDN1 or siCtrl for 6 h, and then treated with gefitinib (1 μM) for another 48 h, and the expression levels of claudin1, Oct4, Sox2, and CD133 were evaluated.

We established a lentivirus-ShRNA-CLDN1 carrying an enhanced green fluorescent protein (ZsGreen) and determined the best infection conditions for PC9/GR cells (Fig. 3A). The xenograft mice model was developed as shown in Fig. 3B. The combination of shCLDN1 and gefitinib significantly suppressed tumor progression, when compared to any single treatment (Fig. 3C–E). Moreover, the combination of claudin1 knockdown and gefitinib significantly suppressed claudin1 expression, when compared to gefitinib administration alone (Fig. 4F, G and Supplemental Fig. Sb). The effects of claudin1 downregulation in decreasing gefitinib resistance have been documented in xenograft mouse models.

### Claudin1 knockdown inhibits cancer stem-like properties in NSCLC cells

Claudin1 knockdown decreased the transcript levels of pluripotent markers (Fig. 4A). Then, the best lentivirus-ShRNA-CLDN1 infection conditions for H1975 cells were determined (Fig. 4B). Claudin1 knockdown in PC9/GR and H1975 cells significantly inhibited protein expression levels of pluripotent markers (Fig. 4C, D and Supplemental Fig. S2A, B and ScA, B). Consistently, claudin1 knockdown significantly inhibited sphere formation (Fig. 4E, F). The combination of knocking-down claudin1 and gefitinib administration suppressed the expression levels of pluripotent markers when compared to gefitinib administration alone in PC9/GR cells (Fig. 4G and Supplemental Fig. S2C–G and ScC) and in H1975 cells (Fig. 4H and Supplemental Fig. S2H–K and ScD). These data suggest that one mechanism through which claudin1 downregulation decreases gefitinib resistance is *via* the association between claudin1 and cancer cell stemness.

### AKT activation participate claudin1-mediated cancer stem-like properties in NSCLC cells

**Fig. 5:**
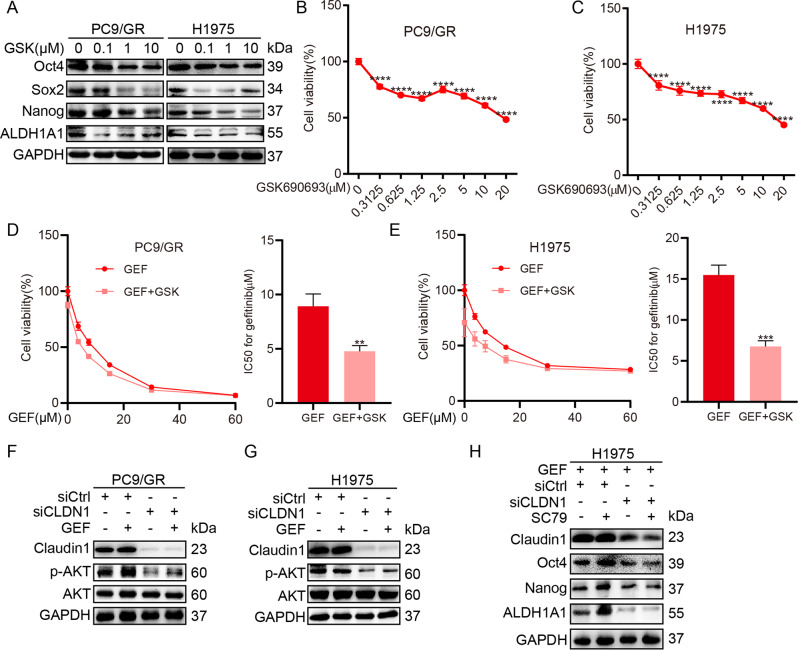
AKT activation mediates the relevance between cluadin1 and CSC-like properties. **A** The expression levels of claudin1 and pluripotent markers were detected after PC9/GR and H1975 cells had been treated with GSK690693 for 48 h. **B**, **C** Effects of indicated concentrations of GSK690693 on cell viability of PC9/GR and H1975 cells. ****P < 0.0001 *vs* GSK690693 (0 µM). **D**, **E** Effects of GSK690693 (0.2 μM) on gefitinib resistance in PC9/GR and H1975 cells as detected by the CCK8 assay, and the IC50 against gefitinib was determined. **P < 0.01, ***P < 0.001. **F**, **G** PC9/GR and H1975 cells were transfected with siCLDN1 or siCtrl for 48 h, then treated with gefitinib (1 μM) for 30 min, and expression levels of claudin1, p-AKT, and AKT were determined by western blotting. **H** H1975 cells were transfected with siCLDN1 or siCtrl for 6 h, then cells were treated with gefitinib (1 μM) and SC79 (2 μM) for another 48 h after which the expression levels of claudin1, Oct4, Nanog, and ALDH1A1 were determined.

We found that GSK690693, an AKT kinase inhibitor, suppressed the expression levels of Oct4, Sox2, Nanog, and ALDH1A1 (Fig. 5A and Supplemental Fig. S3A, B and SdA), induced growth inhibition (Fig. 5B, C), and decreased gefitinib resistance (Fig. 5D, E). Notably, claudin1 knockdown inhibited p-AKT expression while the combination of claudin1 loss and gefitinib treatment suppressed the expression levels of p-AKT, when compared to gefitinib alone (Fig. 5F, G and Supplemental Fig. S3C, D and SdB-C). In addition, SC79, an AKT phosphorylation activator, induced the expression levels of Oct4, Nanog, and ALDH1A1; however, it had no significant effect on claudin1 expression. Claudin1 knockdown reversed SC79 induced expression of these proteins (Fig. 5H and Supplemental Fig. S3E and SdD). These results suggest that claudin1 downregulation reduces cancer cell stemness by inhibiting AKT activation.

### 1,25(OH)_2_D_3_ inhibits claudin1/AKT/cancer cell stemness pathway by suppressing β-catenin

**Fig. 6:**
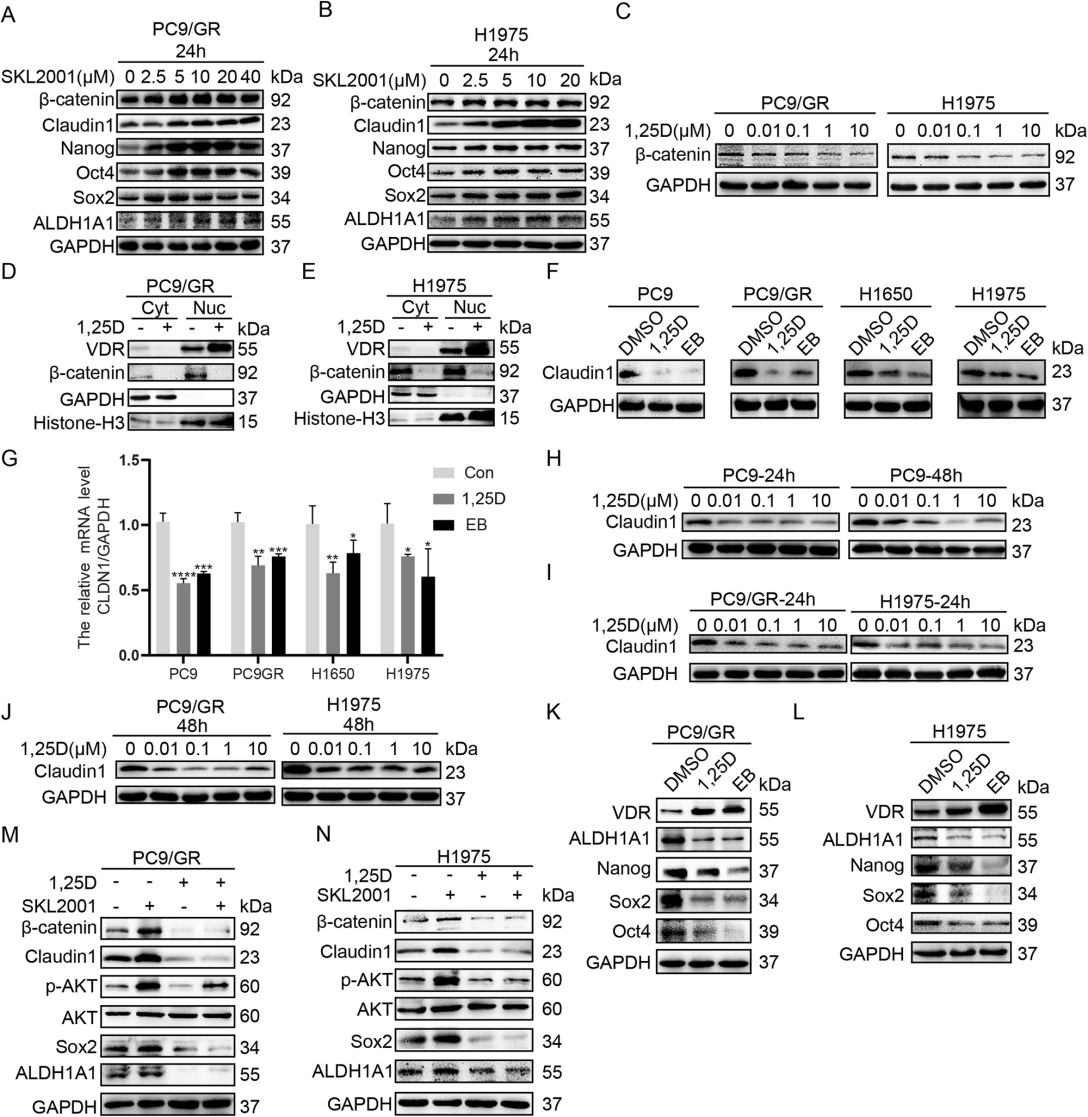
1,25D inhibits claudin1/AKT/cancer cell stemness pathway by inhibiting β-catenin activation. **A**, **B** PC9/GR and H1975 cells were stimulated with various concentrations of SKL2001 for 24 h, then the expression levels of claudin1, Nanog, Oct4, Sox2, and ALDH1A1 were determined. **C** PC9/GR and H1975 cells were treated with various concentrations of 1,25D after which β-catenin level was determined by western blotting. **D**, **E** Nuclear and cytoplasmic fractions of PC9/GR and H1975 cells in the presence or absence of 1,25D were prepared, and VDR and β-catenin expression levels were determined by western blotting. GAPDH and Histone-H3 were, respectively, used as cytoplasm and nucleus loading controls. **F** Expression levels of claudin1 were determined after PC9, PC9/GR, H1975, and H1650 cells were treated with DMSO, 1,25D (100 nM), or EB (100 nM) for 48 h. **G** Relative mRNA expression levels of *CLDN1* were detected by real-time PCR after PC9, PC9/GR, H1650, and H1975 cells had been treated with 1,25D or EB (mean ± SD; *n* = 6; *P < 0.05, **P < 0.01, ***P < 0.001, ****P < 0.0001). **H**–**J** PC9, PC9/GR, and H1975 cells were treated with various concentrations of 1,25D, respectively, for 24 and 48 h after which claudin1 protein expression was evaluated. **K**, **L** PC9/GR and H1975 cells were treated with DMSO, 1,25D (100 nM), or EB (100 nM) for 48 h, then the expression levels of VDR, ALDH1A1, Nanog, Oct4, and Sox2 were evaluated by western blotting. **M**, **N** PC9/GR and H1975 cells were, respectively, treated with 1,25D, SKL2001, or a combination of 1,25D and SKL2001, and the expression levels of β-catenin, claudin1, p-AKT, Sox2, and ALDH1A1 were determined.

Next, we investigated the mechanisms involved in the regulation of claudin1/AKT/cancer cell stemness pathway. We found that SKL2001, an agonist of Wnt/β-catenin signaling pathway, which could stabilize nuclear β-catenin expression [23], increased significantly the expression levels of β-catenin, claudin1 as well as pluripotent markers in PC9/GR and H1975 cells (Fig. 6A, B, Supplemental Fig. S4 and SeA, B). Moreover, 1,25(OH)_2_D_3_ (1,25D) suppressed protein expression of β-catenin in PC9/GR and H1975 cells (Fig. 6C and Supplemental Fig. S5A, B and SeC), enhanced VDR expression while inhibiting β-catenin expression in the nucleus (Fig. 6D, E and Supplemental Fig. SeD, E). Interestingly, protein and mRNA expression levels of claudin1 were suppressed after treatment of NSCLC cells with 1,25D or EB1089 (EB) (a synthetic analog of 1,25D) (Fig. 6F, G and Supplemental Fig. SfA). Moreover, 1,25D treatment for 24 h and 48 h reduced claudin1 expression in PC9, PC9/GR, and H1975 cells (Fig. 6H–J and Supplemental Fig. SfB–D). In addition, 1,25D and EB significantly increased the expression levels of VDR and suppressed the expression levels of pluripotent markers (Fig. 6K, L and Supplemental Fig. S5C, D and SgA, B). Furthermore, SKL2001-mediated upregulation of expression levels of claudin1, p-AKT and pluripotent markers was weakened by 1,25D treatment (Fig. 6M, N, Supplemental Fig. S5E, F and SgC, D). These findings suggest that β-catenin activation-induced expression upregulation of claudin1, p-AKT, and pluripotency markers could be reversed by 1,25D.

### 1,25(OH)_2_D_3_ inhibits cancer stem-like properties and gefitinib resistance by suppressing claudin1 expression and AKT activation

**Fig. 7:**
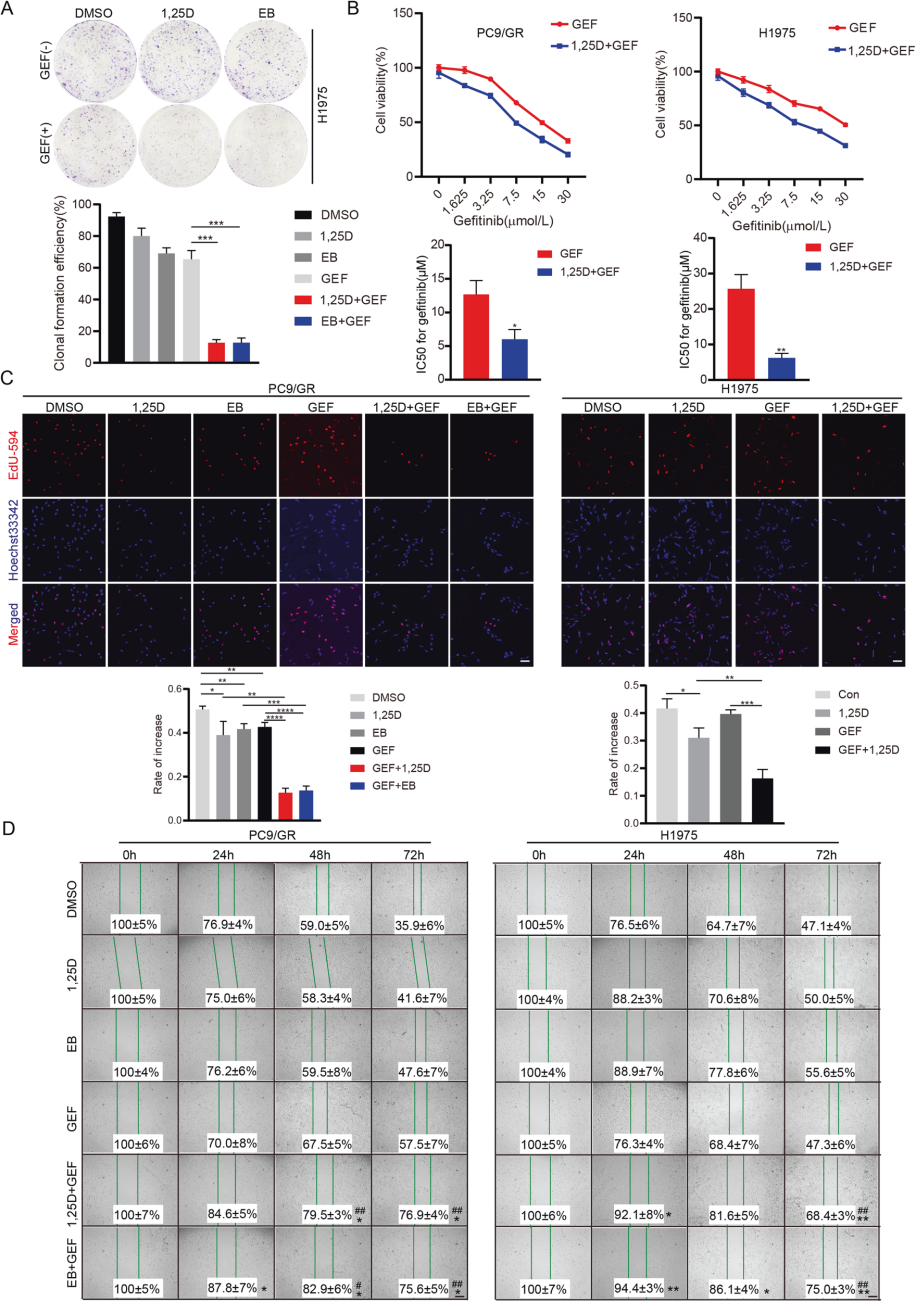
1,25D inhibits gefitinib resistance in NSCLC cells. **A** H1975 cells were seeded into 6-well plates (*n* = 3 per group). The next day, the cells were treated with vehicle (control), 1,25D alone (100 nM), EB (100 nM), gefitinib (1 μM), or with a combination of gefitinib and 1,25D or EB. Treatments were repeated every 3 days. Colony formation was assessed by crystal violet staining. Colony numbers were counted by using the ImageJ software and clonal formation efficiency was calculated (mean ± SD; *n* = 3; ***P < 0.001). **B** PC9/GR and H1975 cells were exposed to various concentrations of gefitinib and gefitinib + 1,25D (100 nM) for 2 days. Cell proliferation was determined by the CCK8 assay. The IC50 value against gefitinib was analyzed. Data are presented as mean ± SD (*n* = 3, *P < 0.05, **P < 0.01). **C** PC9/GR and H1975 cells were exposed to vehicle, 1,25D (100 nM), EB (100 nM), gefitinib (1 μM), 1,25D + gefitinib, or EB + gefitinib for 48 h and stained for Edu (Scale bar: 100 μm; original magnification: ×100; representative images from three experiments). Cell proliferation rate was calculated as a percentage of Edu-positive nuclei to total nuclei (mean ± SD; *n* = 3; *P < 0.05, **P < 0.01, ***P < 0.001, ****P < 0.0001). **D** Horizontal migration of PC9/GR and H1975 cells (gap-closing assay) was evaluated after cells had been treated with the vehicle, 1,25D (100 nM), EB (100 nM), gefitinib (1 μM), 1,25D + gefitinib, or EB + gefitinib at the indicated time points, and the migration rate was calculated (mean ± SD; *n* = 3; ^#^*P* < 0.05, ^##^*P* < 0.01 vs 1,25D or EB; *P < 0.05, **P < 0.01 vs GEF).

**Fig. 8:**
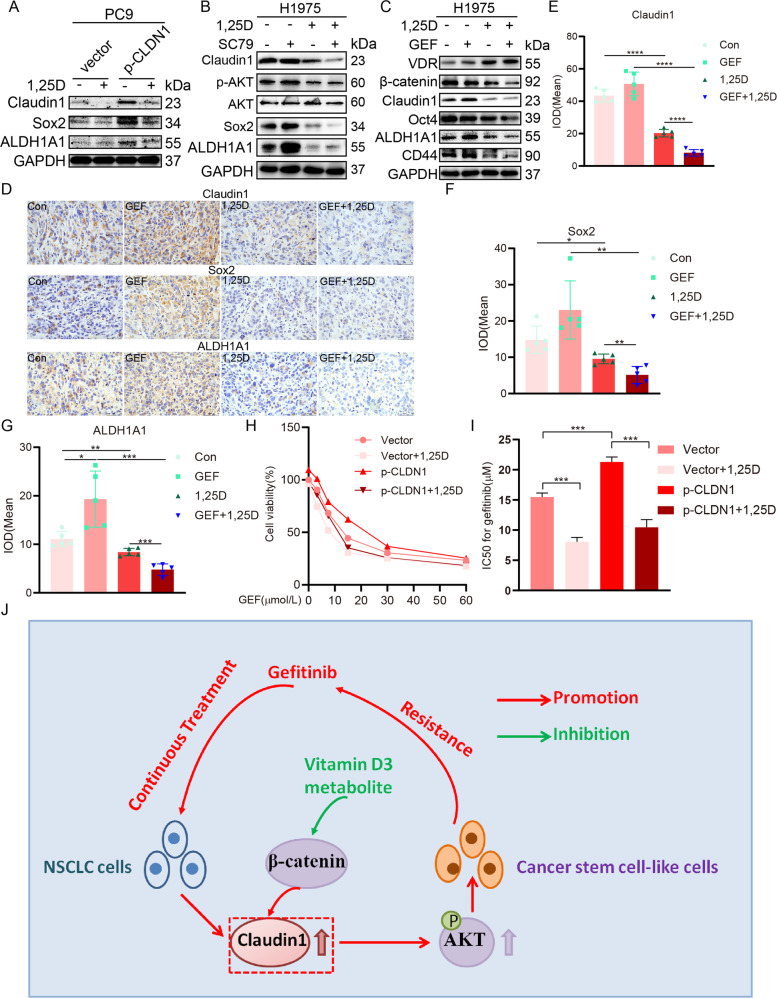
1,25D inhibits cancer cell stemness and gefitinib resistance by suppressing claudin1/AKT pathway. **A** PC9 cells overexpressing claudin1 were treated with or without 1,25D and the expression levels of claudin1, Sox2, and ALDH1A1 were determined. **B** H1975 cells were treated with SC79, 1,25D, and the combination of SC79 and 1,25D for 48 h, then the expression levels of β-catenin, claudin1, p-AKT, Sox2, and ALDH1A1 were determined by western blotting. **C** H1975 cells were treated with 1,25D (100 nM), gefitinib (1 μM), or 1,25D (100 nM) + gefitinib (1 μM) for 48 h. Then the expression levels of VDR, β-catenin, claudin1, Oct4, ALDH1A1, and CD44 were determined by western blotting. **D** Expression levels of claudin1, Sox2, and ALDH1A1 in tumor tissues of each indicated group were detected by immunohistochemistry. **E**–**G** Mean IOD of claudin1, Sox2, and ALDH1A1 expression was analyzed by the IPP software (scale bar: 50 μm; original magnification: ×400); *P < 0.05, **P < 0.01, ***P < 0.001, ****P < 0.0001. **H**, **I** H1975 cells were transfected with pcDNA-3.1/CLDN1 or the empty vector and treated with 1,25D (100 nM) or various concentrations of gefitinib for 48 h. Then cell viability and IC50 against gefitinib were calculated. ***P < 0.001. **J** A model of claudin1 contributes to gefitinib resistance: continuous exposure to gefitinib upregulates claudin1 expression, which promotes CSC-like properties of NSCLC cells and gefitinib resistance by activating the AKT pathway. 1,25(OH)_2_D_3_ reduces expression levels of claudin1, p-AKT, and pluripotency markers by inhibiting β-catenin and suppressing claudin1 and AKT activation-mediated cancer stem-like properties and gefitinib resistance.

Next, we determined whether 1,25D inhibited cancer stem-like properties and gefitinib resistance by regulating claudin1 and AKT activation. We found that 1,25D and EB alleviated gefitinib resistance by inhibiting cell clone formation (Fig. 7A), and by reducing cell viability (Fig. 7B), proliferation (Fig. 7C), and migration (Fig. 7D). Claudin1 overexpression-induced elevations in expression levels of Sox2 and ALDH1A1 were reversed by 1,25D treatment in PC9 cells (Fig. 8A and Supplemental Fig. S6A and ShA). In addition, SC79 induced the expression levels of p-AKT, Sox2, and ALDH1A1, however, it had no significant effects on the expression of claudin1. Notably, 1,25D inhibited claudin1 expression and reversed the SC79 induced upregulated expression levels of p-AKT, Sox2, and ALDH1A1 (Fig. 8B, Supplemental Fig. S6B and ShB). The combination of 1,25D and gefitinib significantly increased VDR expression while reducing the expression levels of β-catenin, claudin1, and stemness markers, when compared to gefitinib treatment alone (Fig. 8C, Supplemental Fig. S6C and ShC). Expression levels of claudin1, Sox2, and ALDH1A1 in tumor tissues of 1,25D/gefitinib combination treatment group were decreased when compared to the single treatment group (Fig. 8D–G). Furthermore, claudin1 overexpression-induced increase in gefitinib resistance in H1975 cells was reversed by 1,25D treatment (Fig. 8H, I). These results indicate that 1,25D inhibits gefitinib resistance by inhibiting claudin1 and AKT activation mediated cancer cell stemness.

## Discussion

In this study, we investigate the relationship between claudins and EGFR-TKI resistance in NSCLC cell lines, which has not been reported previously. Our study demonstrates that claudin1 expression is positively correlated with EGFR-TKI resistance, and claudin1 knockdown suppresses gefitinib resistance by inhibiting AKT activation-mediated cancer stem-like properties. Furthermore, 1,25D treatment reduces claudin1 expression, AKT activation, and cancer cell stemness by inhibiting β-catenin and reverses claudin1 and AKT activation mediated-cancer stem-like properties and gefitinib resistance (Fig. 8J).

Based on GEO datasets analysis, we found that claudin1, which was one of the most dysregulated claudins in human cancers [8], played an important role in regulating EGFR-TKI resistance. For the first time, we reported that claudin1 expression was upregulated in EGFR-TKI resistant NSCLC cells. Interestingly, gefitinib treatment significantly increased the expression level of claudin1 in gefitinib-resistant NSCLC cells. Moreover, long-term gefitinib treatment induced claudin1 expression in gefitinib-sensitive NSCLC cells, indicating the important role of gefitinib in the upregulation of claudin1 expression during resistance induction. By knocking down claudin1 expression in vitro and in vivo, we established that downregulation of claudin1 decreased gefitinib resistance. These findings underscore the importance of claudin1 as a predictor of EGFR-TKI resistance and may be a promising target for the treatment of EGFR-TKI resistant NSCLC.

Resistance to EGFR-TKIs induces a cancer stem cell phenotype while suppression of cancer stem cell properties ameliorates EGFR-TKIs resistance [24, 25]. ALDH1A1-positive lung cancer cells were shown to exhibit resistance to gefitinib when compared to ALDH1A1-negative lung cancer cells [26]. LUAD resistance to EGFR-TKIs therapy is associated with increased expression levels of Sox2, Oct4, and Nanog [12]. In addition, inhibition of stem cell-like properties or knockdown of stem cell-related markers restores EGFR-TKIs cytotoxicity [11, 27]. Notably, we found that claudin1 downregulation inhibited the expression levels of Oct4, Nanog, Sox2, and ALDH1A1, as well as the ability for sphere formation. In summary, claudin1 downregulation may inhibit gefitinib resistance by suppressing cancer stem-like properties. Claudin1 mediated enrichment of cancer stem-like cells provides a new axis-of-evil for preferential therapeutic targeting of EGFR-TKI resistance, which has potential clinical consequences.

The AKT pathway is a major downstream effector of EGFR signaling and has been implicated in cell survival. Pro‐survival AKT is active in gefitinib‐resistant EGFR mutant NSCLC cells [28]. Activation of AKT signaling is a convergent feature in NSCLC patients and an EGFR mutation with acquired resistance to EGFR-TKIs may be due to multiple underlying mechanisms [29, 30]. Moreover, p-AKT expression levels are found to be upregulated in EGFR-TKI resistant cell lines compared to their corresponding parental cell lines [31, 32], and upregulation of p-AKT confers EGFR-TKI resistance in EGFR-mutant lung cancers [33, 34]. We found that GSK690693 inhibited gefitinib resistance and expression levels of claudin1, Oct4, Sox2, Nanog, and ALDH1A1. Notably, claudin1 loss reduced the expression of p-AKT in PC9/GR and H1975 cells. Moreover, claudin1 downregulation suppressed AKT activation-mediated cancer cell stemness. Therefore, claudin1 downregulation suppressed cancer cell stemness and gefitinib resistance by inhibiting AKT activation.

Recently, more and more research focus on the role of β-catenin in regulating EGFR-TKI resistance. β-catenin signaling mediates EGFR-TKI resistance in EGFR mutant NSCLC [35–37], and activation of β-catenin signaling pathway induces EGFR-TKI resistance in NSCLC cells [36–38]. In addition, inhibition of β-catenin decreases stem cell-like properties and enhances anticancer effects of EGFR-TKIs in EGFR-mutated non-small-cell lung cancer [39, 40]. Inhibition of β-catenin significantly reduces tumor burdens and improves recurrence-free survival as well as overall survival outcomes in xenograft models of EGFR-TKI-resistant NSCLC cells [35]. Epidemiological studies indicate that vitamin D insufficiency has an etiological role in various human cancers. The active metabolite of vitamin D, 1,25(OH)_2_D_3_, or vitamin D analogs are potential anticancer agents, since their administrations have antiproliferative effects, can activate apoptotic pathways, and inhibit angiogenesis [41, 42]. Notably, 1,25(OH)_2_D_3_ was shown to inactivate the β-catenin pathway in colorectal cancer [43, 44] and in breast cancer cells [45]. In this study, we found that β-catenin activation upregulated the expression levels of claudin1, p-AKT, and pluripotency markers in EGFR mutant NSCLC cells, while 1,25D treatment suppressed expression levels of β-catenin, claudin1, p-AKT, and pluripotency markers. Furthermore, 1,25D suppressed the expression levels of claudin1, p-AKT and pluripotency markers by inhibiting β-catenin, and by forcing claudin1 expression and activating AKT, we further determined that 1,25(OH)_2_D_3_ may reduce cancer cell stemness and suppress gefitinib resistance by inhibiting claudin1/AKT pathway.

In summary, we uncovered a novel mechanism involved in gefitinib resistance. Increased claudin1 expression, induced by continuous gefitinib treatment, was involved in acquired EGFR-TKI resistance by promoting AKT activation-mediated enhancement of cancer stem-like properties. 1,25(OH)_2_D_3_ suppressed expression levels of claudin1, p-AKT, and pluripotency markers by inhibiting β-catenin thereby suppressing gefitinib resistance. Therefore, targeting β-catenin/claudin1/AKT signaling is a potential therapeutic strategy for enhancing the efficacy of EGFR-TKIs in patients with acquired resistance.

## Materials and methods

### Cell lines

The NSCLC lines, PC-9 and PC9/GR with EGFR exon 19 deletion [delE746-A750], without T790M mutations and MET gene amplification (kind gifts from Dr. Zhou Caicun, Shanghai pulmonary hospital, Shanghai, China), HCC827 (a gift from Peking Union Medical College), H1975 with EGFR L858R/T790M mutation (a kind gift from 3D Medicines, Shanghai, China), and H1650 with EGFR exon 19 deletion and PTEN loss (a kind gift from 3D Medicines, Shanghai, China) [46] were cultured in Dulbecco’s modified Eagle’s medium (DMEM) (Biological Industries, Kibbutz Beit-Haemek, Israel) or RPMI 1640 (Biological Industries) supplemented with 10% fetal bovine serum (FBS) (Biological Industries), 100 μg/mL streptomycin and 100 U/mL penicillin (KeyGEN BioTECH, Nanjing, China) in a humidified cell incubator at 37 °C with an atmosphere of 5% CO_2_.

### Reagents and chemicals

The primers used in this study were procured from Genscript (Nanjing, China); Gefitinib, 1,25(OH)_2_D_3_ (calcitriol), and EB 1089 (Seocalcitol) were purchased from Selleck (Shanghai, China); SC79 were obtained from Beyotime Biotechnology (Nantong, China); EGF and FGF-basic were obtained from PeproTech (Rocky Hill, NJ, USA) while B27 was obtained from Gibco (Gaithersburg, MD, USA).

### Western blot analysis

Proteins were extracted from tumor tissues or NSCLC cells by using a lysis buffer (KeyGEN BioTECH, Nanjing, China) and a protease inhibitor cocktail (KeyGEN) for western blotting. Extracted proteins were separated by polyacrylamide SDS gels and electrophoretically transferred onto polyvinylidene fluoride membranes (Millipore, MA, USA). Membranes were probed with indicated antibodies overnight at 4 °C. Antibodies against claudin1 and VDR (Proteintech Group, WUHAN SANYING, WuHan, China, 1:2000 dilution), p-AKT and AKT (Bimake, Houston, TX, USA, 1:1000 dilution), Oct4, Nanog, Sox2, CD133, CD44 and ALDH1A1 (Proteintech Group, 1:1000 dilution), β-catenin (Santa Cruz Biotechnology, Dallas, TX, USA, 1:1000 dilution) and GAPDH (Proteintech Group, 1:10,000 dilution) were used in this study. After washing, the membranes were incubated at room temperature in the presence of a HRP-conjugated goat anti-rabbit IgG secondary antibody (Beyotime) or a goat anti-mouse IgG secondary antibody (AbSci, Vancouver, WA, USA; 1:10,000 dilution) for 1 h. Electrochemical luminescent substrates (Vazyme, Nanjing, China) were used to visualize proteins of interest by using the Tanon imaging system (Tanon, Shanghai, China). Relative expression was quantified densitometrically by using the ChemiScope analysis software and calculated according to reference bands of GAPDH or AKT.

### Immunohistochemistry

Tissues were inflation-fixed, paraffin-embedded, and sliced into 5-μm sections. Sections were used for immunohistochemical examination. Immunohistochemical staining was performed with the following primary antibodies against claudin1, Sox2, and ALDH1A1 (Proteintech group), followed by incubation with horseradish peroxidase (HRP)-conjugated goat anti-rabbit IgG secondary antibody according to manufacturer’s instructions (ZSGB-BIO, Beijing, China). Peroxidase conjugates were subsequently visualized by using a diaminobenzidine (DAB) solution. Then, sections were counterstained with hematoxylin and mounted on coverslips. Between each step, cells were rinsed 3 times for 5 min each time. Staining was photographed by using a Leica microscope (DM2500, Wetzlar, Hesse, Germany). For each mouse, five fields were selected to obtain the average of integrated optical density (IOD) of IHC staining by the Image Pro Plus (IPP) software, and the analysis was done blindly.

### Gene expression dataset

Gene expression datasets from parental and erlotinib-resistant HCC827 cells (accession no. GSE69181) as well as from PC9 and HCC827 cells short-term treated with DMSO and gefitinib (accession no. GSE75308) were downloaded from the Gene Expression Omnibus (GEO) database (https://www.ncbi.nlm.nih.gov/geo/). Platforms of GEO datasets respectively used GPL571 and GPL10558 for the datasets.

### Quantitative real-time PCR

Total RNA was extracted from NSCLC cells by using the Trizol reagent (Vazyme, Nanjing, China) and used for cDNA synthesis (Vazyme). GAPDH mRNA expression levels were used for data normalization. The mRNA primer sequences (GenScript, Nanjing, China) used for QRT-PCR were human CLDN1: 5′-AATCTGAGCAGCACATTG-3′ (forward, F), 5′-GTCTTCCAAGCACTTCATAC-3′ (reverse, R); human POU5F1: 5′-GAGGAAGCTGACAACAATG-3′ (F), 5′-CGGTTCTCGATACTGGTT-3′ (R); human Sox2: 5′-GTGGAAACTTTTGTCGGAGA-3′ (F), 5′-CAGCGTGTACTTATCCTTCT-3′ (R); human NANOG: 5′-CTCCAACATCCTGAACCT-3′ (F), 5′-GTCACACCATTGCTATTCTT-3′ (R); human ALDH1A1: 5′-CTGTCCTACTCACCGATT-3′ (F), 5′-TCTTGCCACTCACTGAAT-3′ (R); human GAPDH: 5′-CTTCTTTTGCGTCGCCAGCCGA-3′ (F), 5′-ACCAGGCGCCCAATACGACCAA-3′ (R). These primers were used to quantify the expression levels (Vazyme) with RT-PCR (Applied Biosystems, Foster City, CA) and results were analyzed with the ΔΔCt method.

### Cell viability assays and Edu staining

Cell viability assays were performed as previously described [47]. Briefly, 5 × 10^3^ cells/well were seeded in 96-well plates. Twenty-four hours after seeding, cells were transfected with siRNA or treated with the indicated drugs for 48 h. Absorbance was measured at the indicated time points. Cell proliferation was quantified based on the incorporation of 5-ethynyl-2’-deoxyuridine (Edu) into DNA by using a BeyoClick™ EdU-594 In Vitro Imaging Kit (Beyotime, Nantong, China) as previously described [48]. A laser scanning confocal microscope (CLSM, Carl Zeiss LSM800) was used to determine the proportion of nucleated cells that had incorporated Edu. The assay was performed in triplicate and repeated three times in independent experiments.

### Colony-formation assay

Cells were treated with various concentrations of gefitinib or transfected with siCtrl or siCLDN1. After 14 days of culture, colonies were fixed in methanol and stained with 0.1% crystal violet (KeyGEN BioTECH, Nanjing, China). Colonies with a diameter greater than 1 mm were counted. Samples were assayed in triplicates.

### Scratch analysis

The scratch assay was used to evaluate the migration ability of PC9/GR and H1975 cells. Briefly, cells were cultured in 6-well plates with a complete medium, grown to full confluence, after which the cell monolayer was scratched with a sterile pipette tip and washed with the medium to remove detached cells. Then, cells were incubated with indicated drugs fully supplemented in the culture medium. The wound gap was monitored under a microscope with corresponding images recorded by using a digital camera. For each image, distances between one side of scratch and the other were quantified at certain intervals (μm) by using the Image Pro Plus software (Media Cybernetics). By comparing the images from 0 h to the indicated time points, the distances of each scratch were obtained and the migration rate was calculated.

### Spheroid colony formation

A total of 5 × 10^3^ cells were seeded into 6-well ultra low-attachment plates (Corning, NY, USA) and incubated in DMEM/F12 (Biological Industries) supplemented with EGF (20 ng/mL), FGF-basic (20 ng/mL), and B27 (20 μL/mL) for 2 weeks. Cell spheres, which were characterized by tight, spherical, non-adherent colonies of >90 μm in diameter, were observed and counted.

### siRNA and plasmid transfection

In all experiments, 150 pmol siRNA (the target sequence of CLDN1‐specific siRNA: 5′‐GCAAAGUCUUUGACUCCUUTT‐3′) (TranSheep Bio and Genomeditech, Shanghai, China) or 5 μg of pcDNA3.1‐CLDN1 or empty vector (TranSheep Bio, Shanghai, China) were used to transfect 70%‐80% confluent cells, according to the manufacturer’s instructions. The Lipofectamine 2000 reagent (Life Technologies, Carlsbad, CA, USA) was used to deliver siRNA or plasmids into PC9/GR, H1975 or PC9 cells growing in serum‐free opti‐MEM media (Gibco, Gaithersburg, MD, USA). After 6 h, the medium containing the siRNA/plasmid–lipid complexes was replaced with DMEM containing 10% FBS. Subsequent experiments were completed at the indicated time after transfection.

### Lentivirus infection

In all, 1.5 × 10^5^ PC9/GR or H1975 cells were seeded in 6-well plates. Twenty-four hours after seeding, cells were treated with polybreen (5 μg/mL) and respectively infected with the various volumes of the lentivirus (3 × 10^8^ TU/mL) carrying shNC or shCLDN1 (Contract number HH20200702WY-LV01, HH20200826WY-LP01, HanBio, Shanghai, China). After 72 h, cells were obtained and ZsGreen positive cells were evaluated by a Laser scanning confocal microscope (CLSM, Carl Zeiss LSM800) (*n* = 2, biological replicates), and the expression levels of claudin1, Nanog, Sox2, Oct4, and ALDH1A1 were evaluated by western blotting and real-time PCR (*n* = 3, biological replicates).

### In vivo mouse model

Four-week-old male BALB/c nude mice were obtained from Yangzhou University (Yangzhou, China). Experiments involving animals were approved by the Ethics Committee of China Pharmaceutical University. Animals were maintained in individual ventilated cages in compliance with institutional guidelines. Xenograft mouse model was established as methods described previously [49]. Briefly, the PC9/GR cells were respectively infected with a lentivirus carrying control shRNA (shNC) or with shCLDN1 (HanBio, Shanghai, China). After 3 days, the cells were harvested and resuspended respectively, then approximately 1.5 × 10^7^ cells were subcutaneously injected into the right forelimbs of mice. The mice of shNC and shCLDN1 groups were randomized into four groups (*n* = 6 per group). When tumor volumes reached an average of 70–100 mm^3^, the mice were treated with gefitinib (50 mg/kg) or drinking water (vehicle) alone. Tumor volumes were measured once every two days and calculated as (length × width^2^)/2. When tumor volumes of the control group reached an average of 800–1000 mm^3^, animals were euthanized. Then xenografts from each group were collected for further analyses. No blinding was carried out for animal experiments.

### Statistical analysis

Data are expressed as mean ± SD. Power analysis was performed for sample size determination. Two-sided statistical tests were performed. Unpaired Student’s *t* test was used for comparisons of means between groups while one-way analysis of variance with Dunnett’s test was used for comparisons of means among multiple groups. Statistical analyses were performed using Prism 8.00 software (GraphPad, San Diego, CA, USA). The differences were considered significant for *P* < 0.05.

## Supplementary information

Supplementary materials

Supplementary materials-Western blot

Author contribution form

## Data Availability

The data generated and/or analyzed during the current study are available from the corresponding author on reasonable request.
