# Supplementary material for: Claudin1 decrease induced by 1,25-dihydroxy-vitamin D3 potentiates gefitinib resistance therapy through inhibiting AKT activation-mediated cancer stem-like properties in NSCLC cells
Source: Cell Death Discov. 2022 Mar 18;8:122. doi: 10.1038/s41420-022-00918-5 (PMC8931006; doi:10.1038/s41420-022-00918-5)
Supplement: Supplementary file 1 — Supplementary materials [file 41420_2022_918_MOESM1_ESM.docx]

**Supplementary materials**


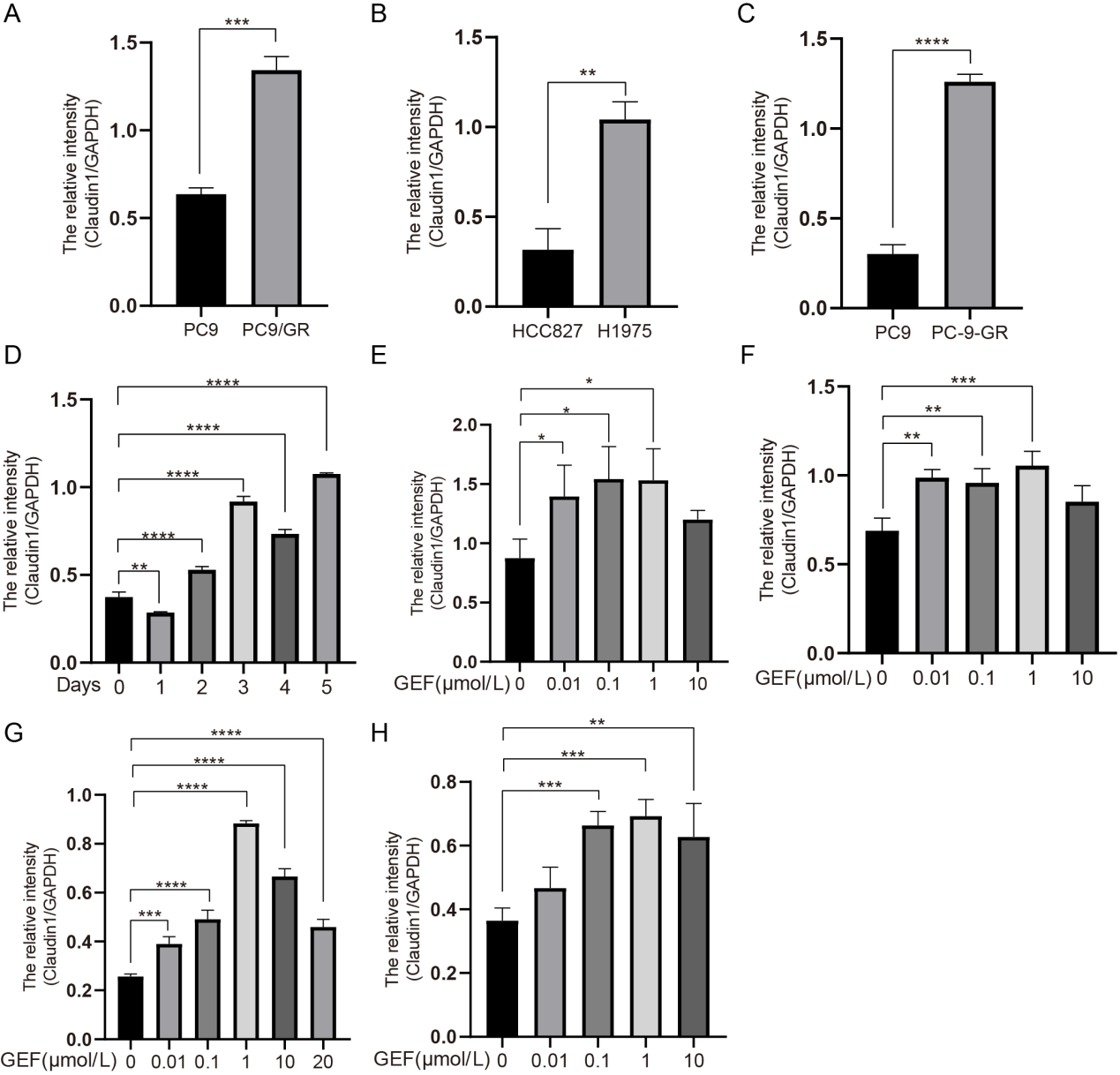


Figure S1. The relative expression of indicated protein in Figure 1D (A-B), Figure 1G (C), Figure 1K (D), Figure 1L (E-F) and Figure 1M (G-H) was quantified by using the ChemiScope analysis software and calculated according to the reference bands of GAPDH (mean ± SD, *n*=3, **P*<0.05, ***P*<0.01, ****P*<0.001, *****P*<0.0001).


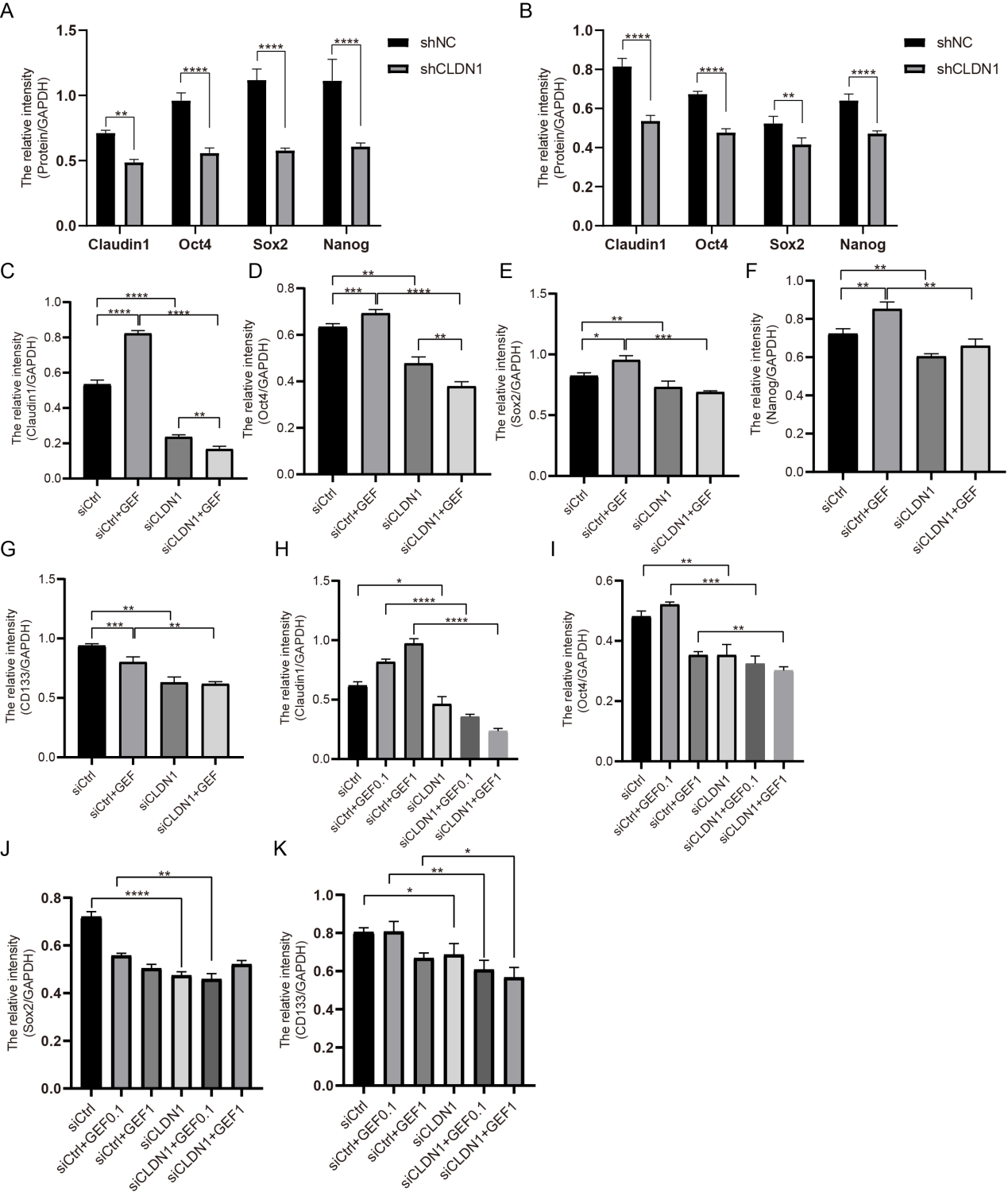


Figure S2. The relative expression of indicated protein in Figure 4C (A), Figure 4D (B), Figure 4G (C-G), and Figure 4H (H-K) was quantified by using the ChemiScope analysis software and calculated according to the reference bands of GAPDH (mean ± SD, *n*=3, **P*<0.05, ***P*<0.01, ****P*<0.001, *****P*<0.0001).


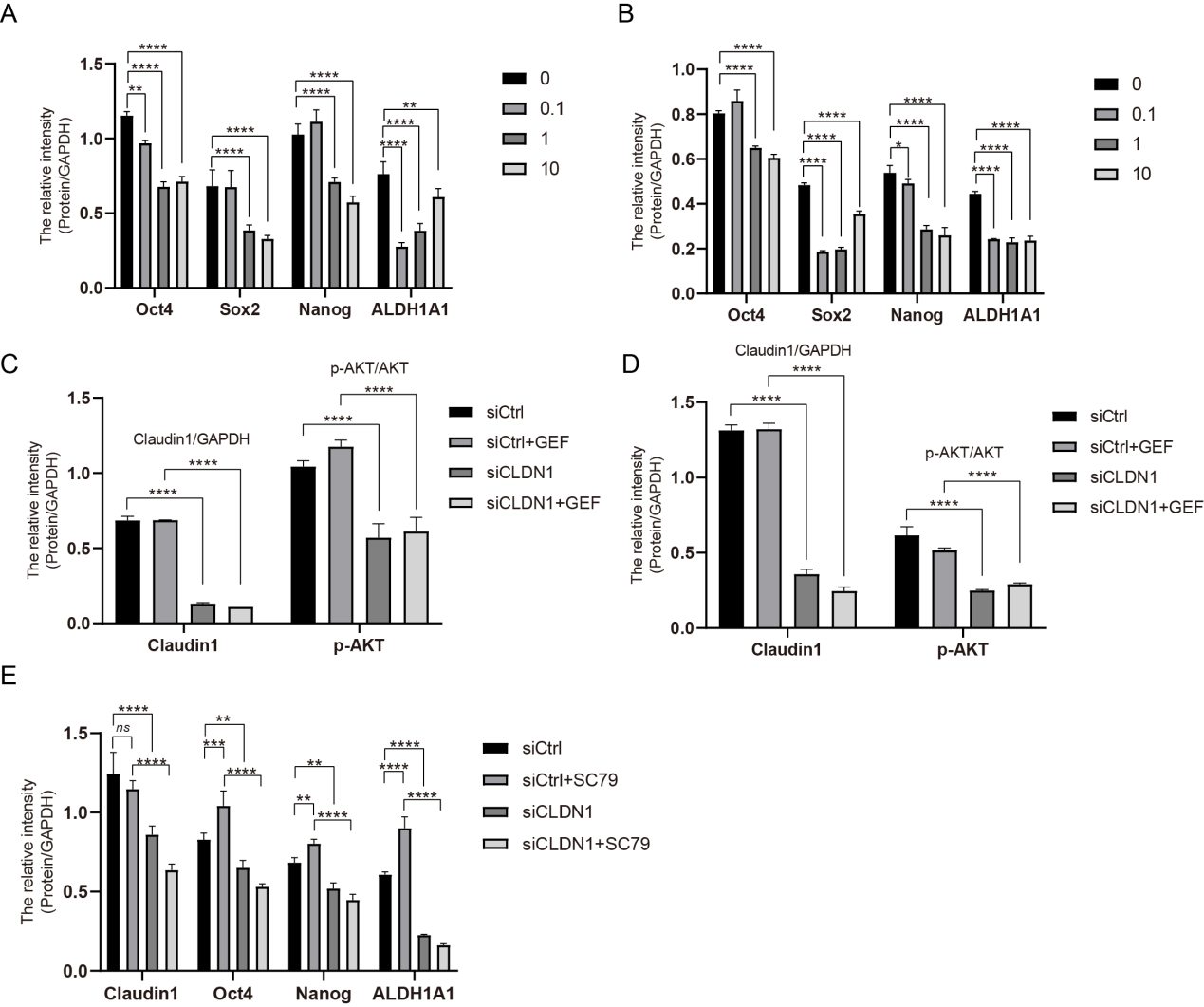


Figure S3. The relative expression of indicated protein in Figure 5A (A-B), Figure 5F (C), Figure 5G (D), and Figure 5H (E) was quantified by using the ChemiScope analysis software and calculated according to the reference bands of GAPDH or AKT (mean ± SD, *n*=3, **P*<0.05, ***P*<0.01, ****P*<0.001, *****P*<0.0001; *ns*: not significant).


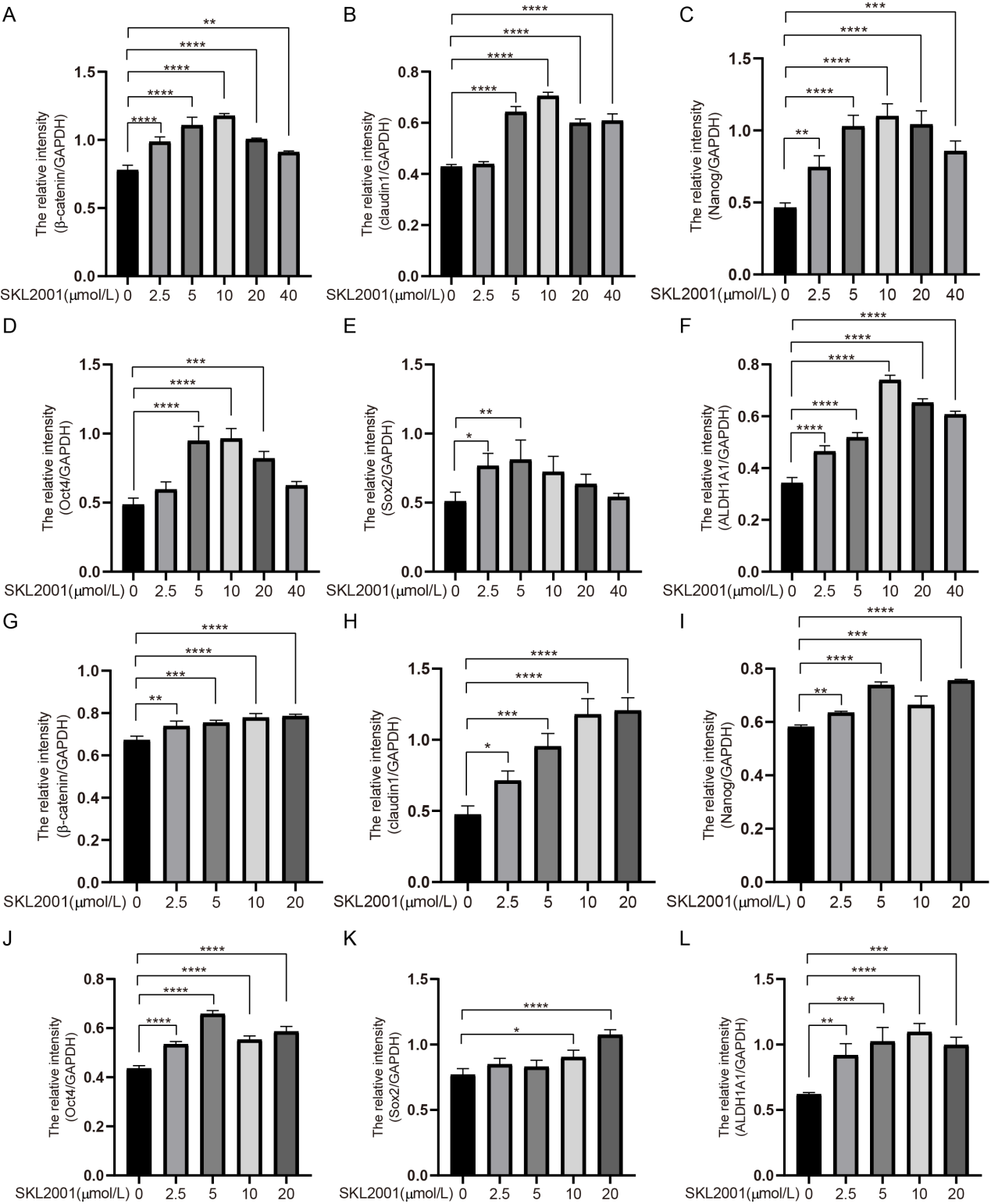


Figure S4. The relative expression of indicated protein in Figure 6A (A-F), and Figure 6B (G-L) was quantified by using the ChemiScope analysis software and calculated according to the reference bands of GAPDH (mean ± SD, *n*=3, **P*<0.05, ***P*<0.01, ****P*<0.001, *****P*<0.0001).


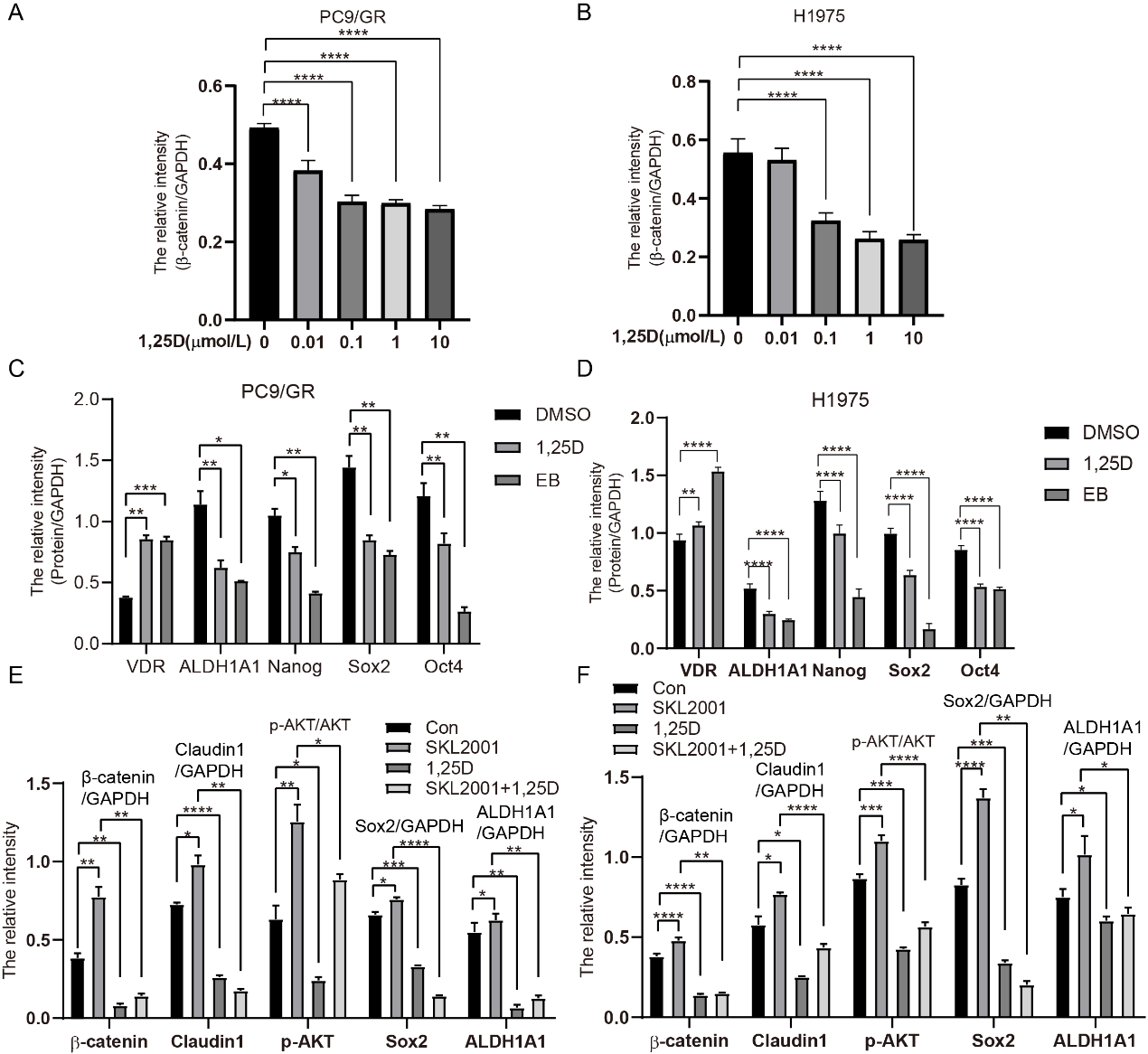


Figure S5. The relative expression of indicated protein in Figure 6C (A-B), Figure 6K (C), Figure 6L (D), Figure 6M (E) and Figure 6N (F) was quantified by using the ChemiScope analysis software and calculated according to the reference bands of GAPDH or AKT (mean ± SD, *n*=3, **P*<0.05, ***P*<0.01, ****P*<0.001, *****P*<0.0001).


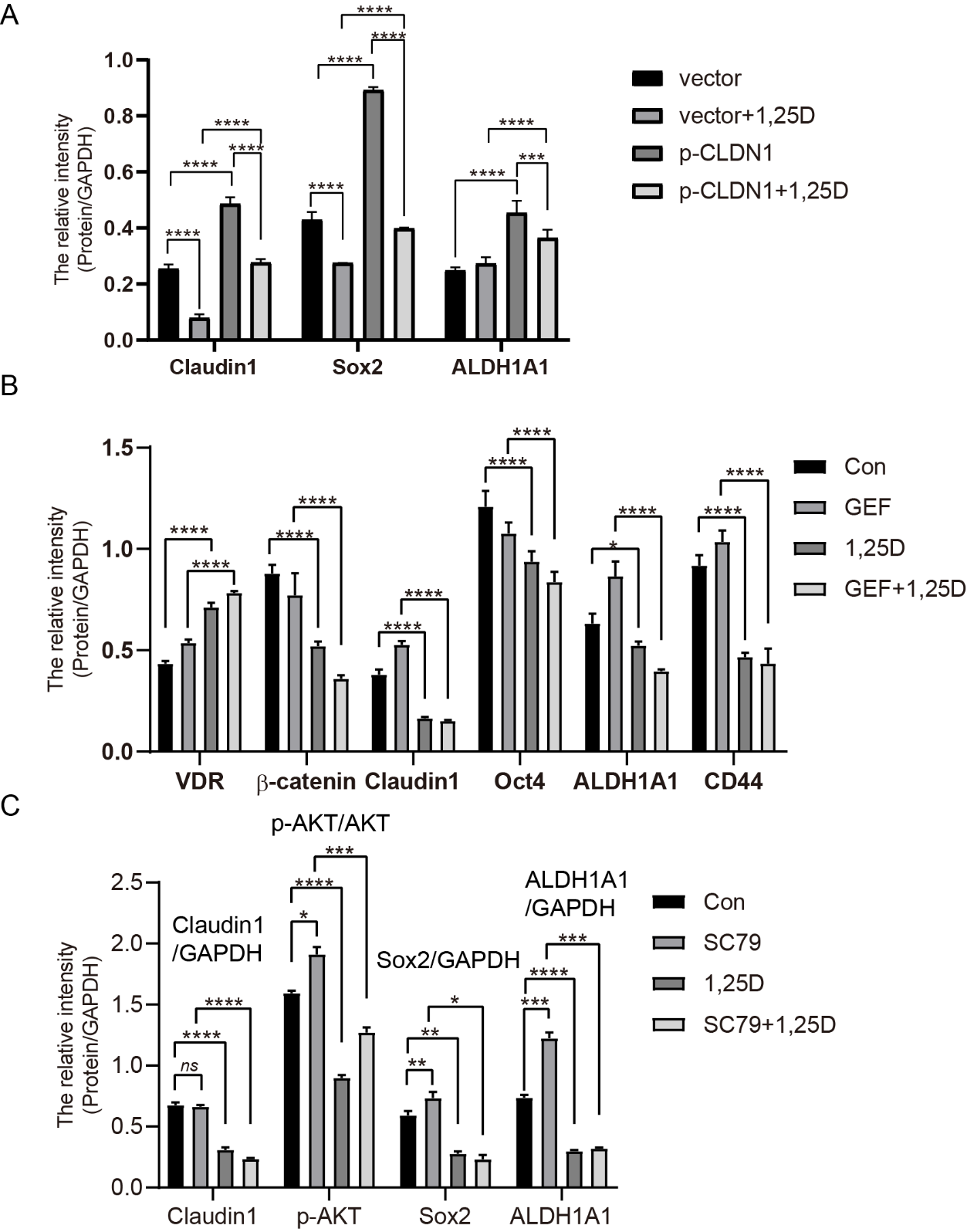


Figure S6. The relative expression of indicated protein in Figure 8A (A), Figure 8B (B), and Figure 8C (C) was quantified by using the ChemiScope analysis software and calculated according to the reference bands of GAPDH or AKT (mean ± SD, *n*=3, **P*<0.05, ***P*<0.01, ****P*<0.001, *****P*<0.0001; *ns*: not significant).
