# Supplementary material for: Claudin1 decrease induced by 1,25-dihydroxy-vitamin D3 potentiates gefitinib resistance therapy through inhibiting AKT activation-mediated cancer stem-like properties in NSCLC cells
Source: Cell Death Discov. 2022 Mar 18;8:122. doi: 10.1038/s41420-022-00918-5 (PMC8931006; doi:10.1038/s41420-022-00918-5)
Supplement: Supplementary file 2 — Supplementary materials-Western blot [file 41420_2022_918_MOESM2_ESM.docx]

**Fig. Sa**


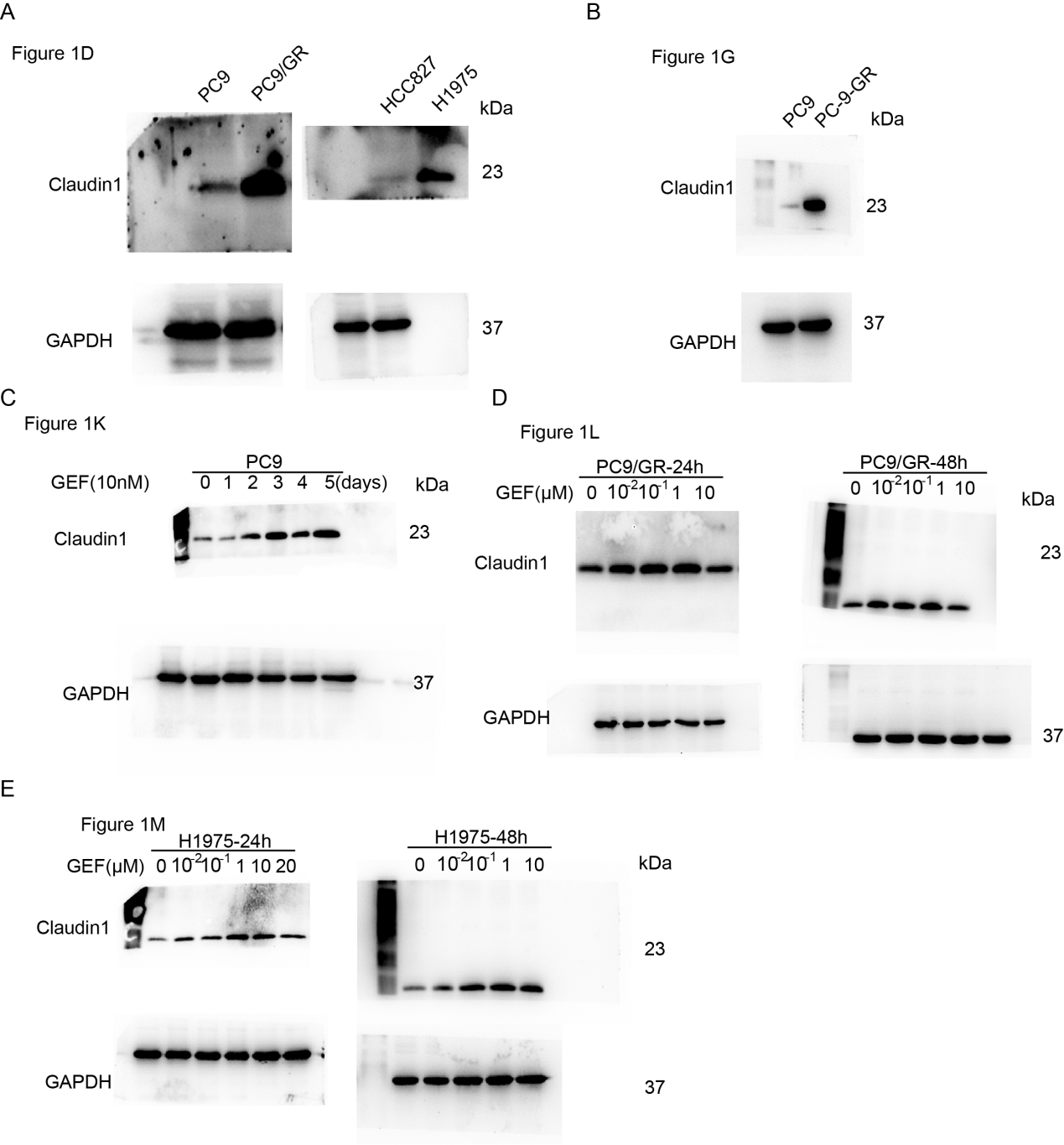


Figure 1 Western blot

**Fig. Sb**


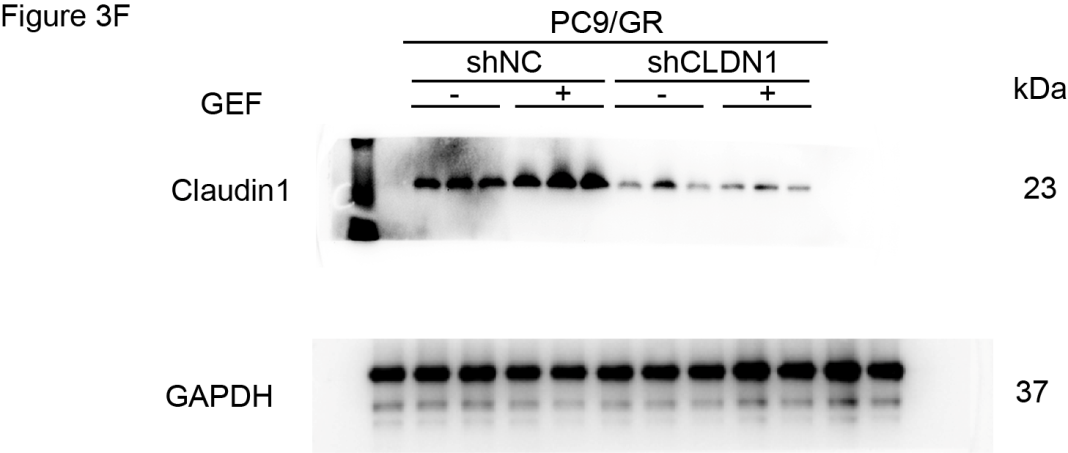


Figure 3 Western blot

**Fig. Sc**


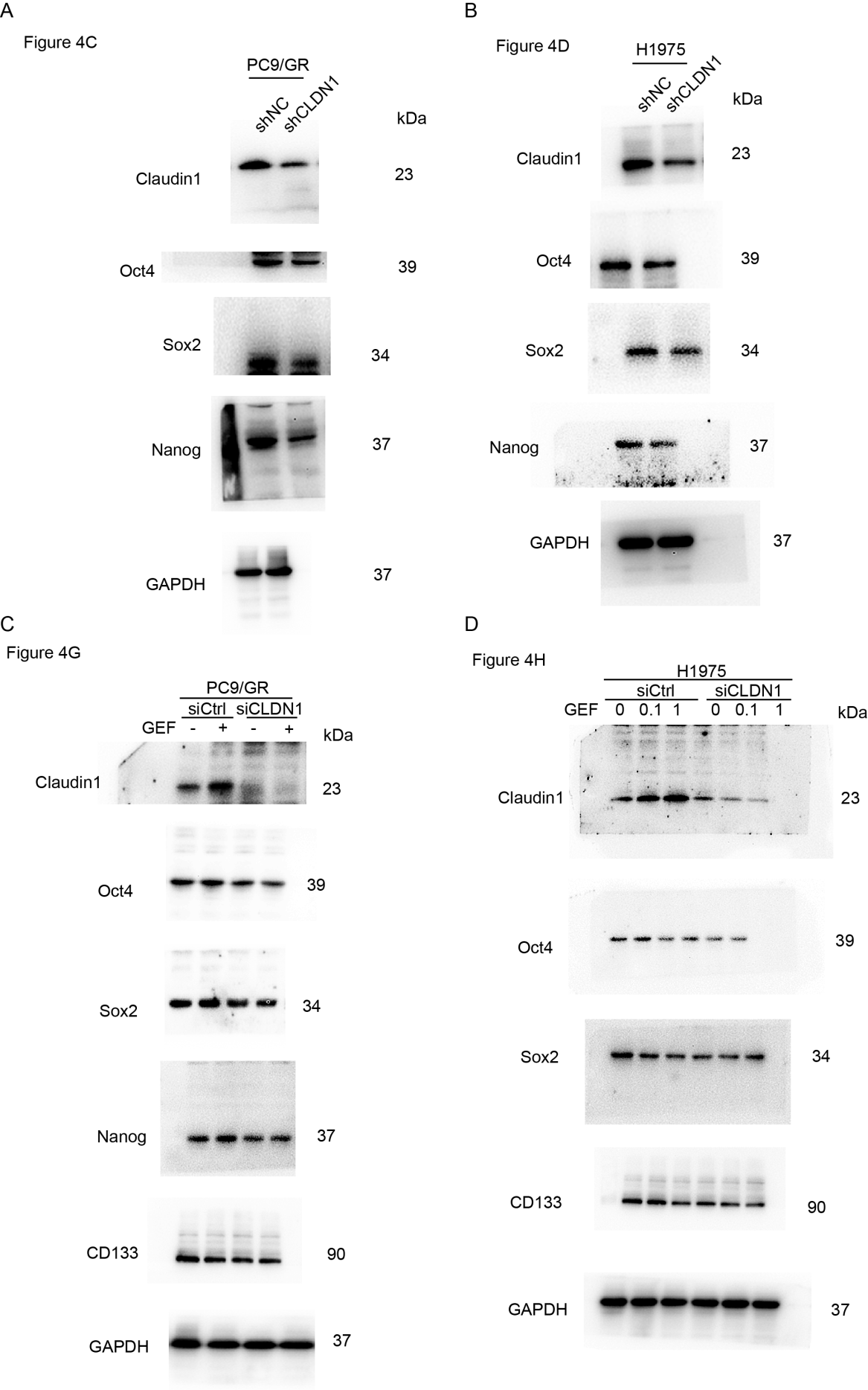


Figure 4 Western blot

**Fig. Sd**


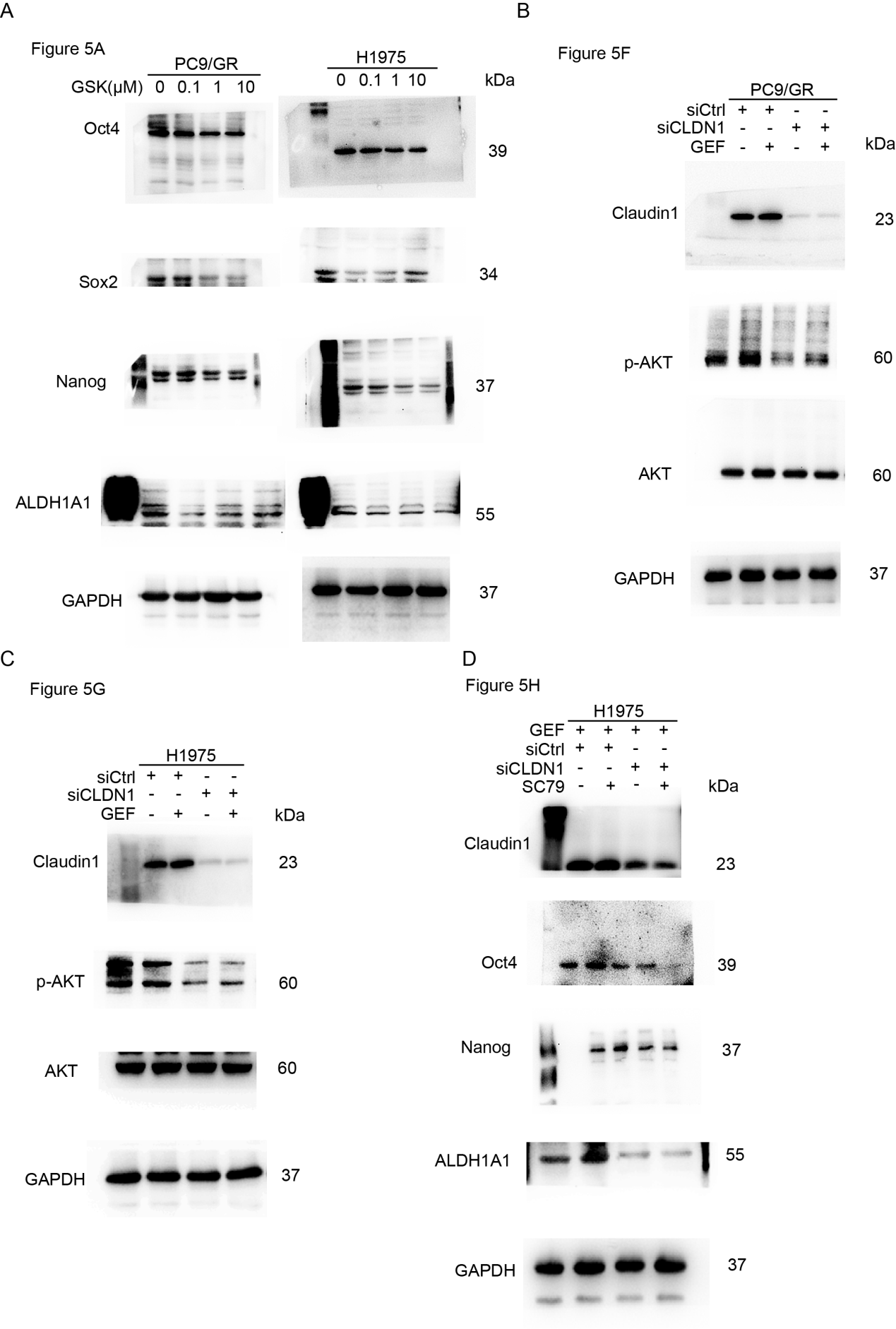


Figure 5 Western blot

**Fig. Se**


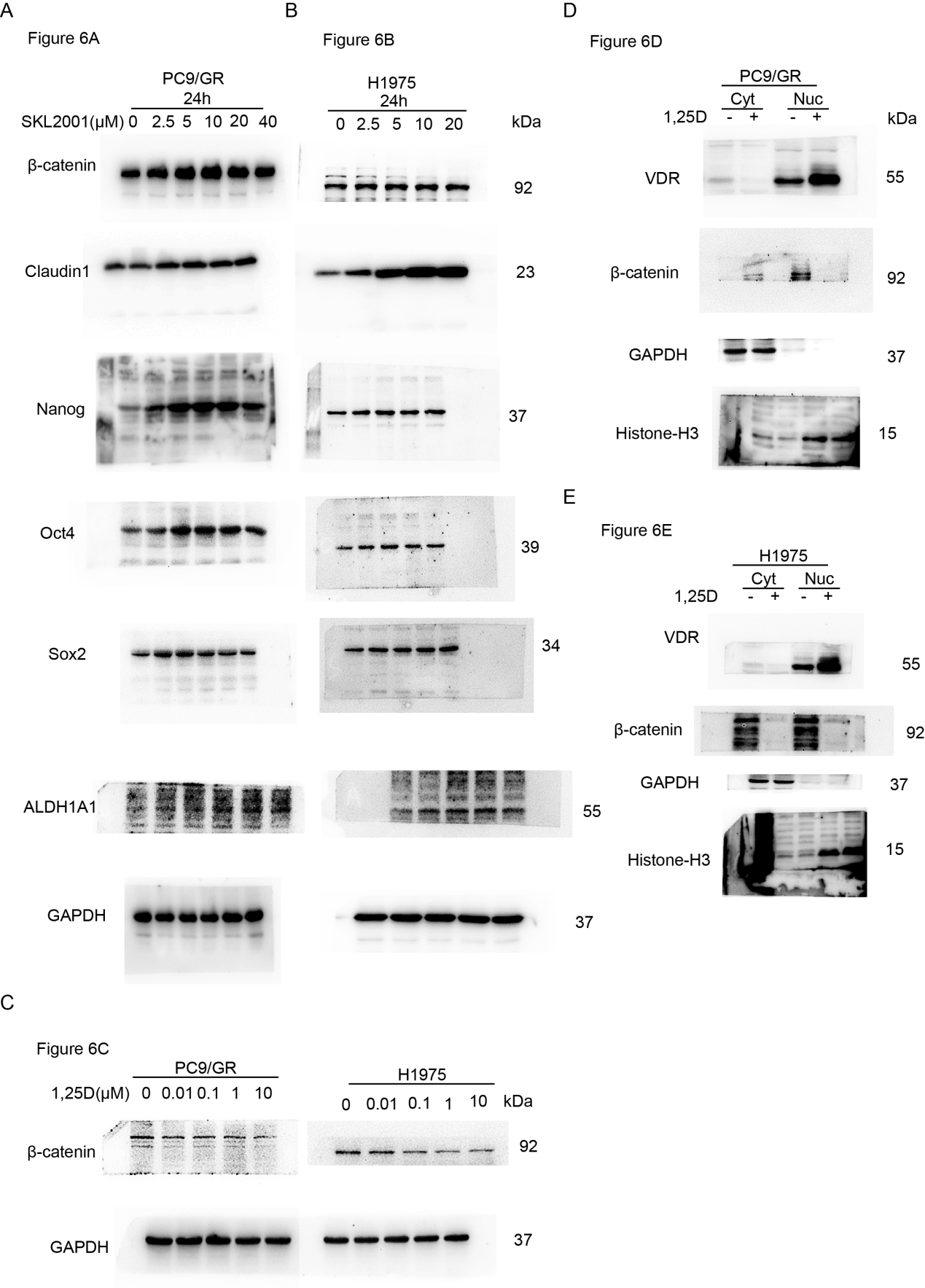


Figure 6-1 Western blot

**Fig. Sf**


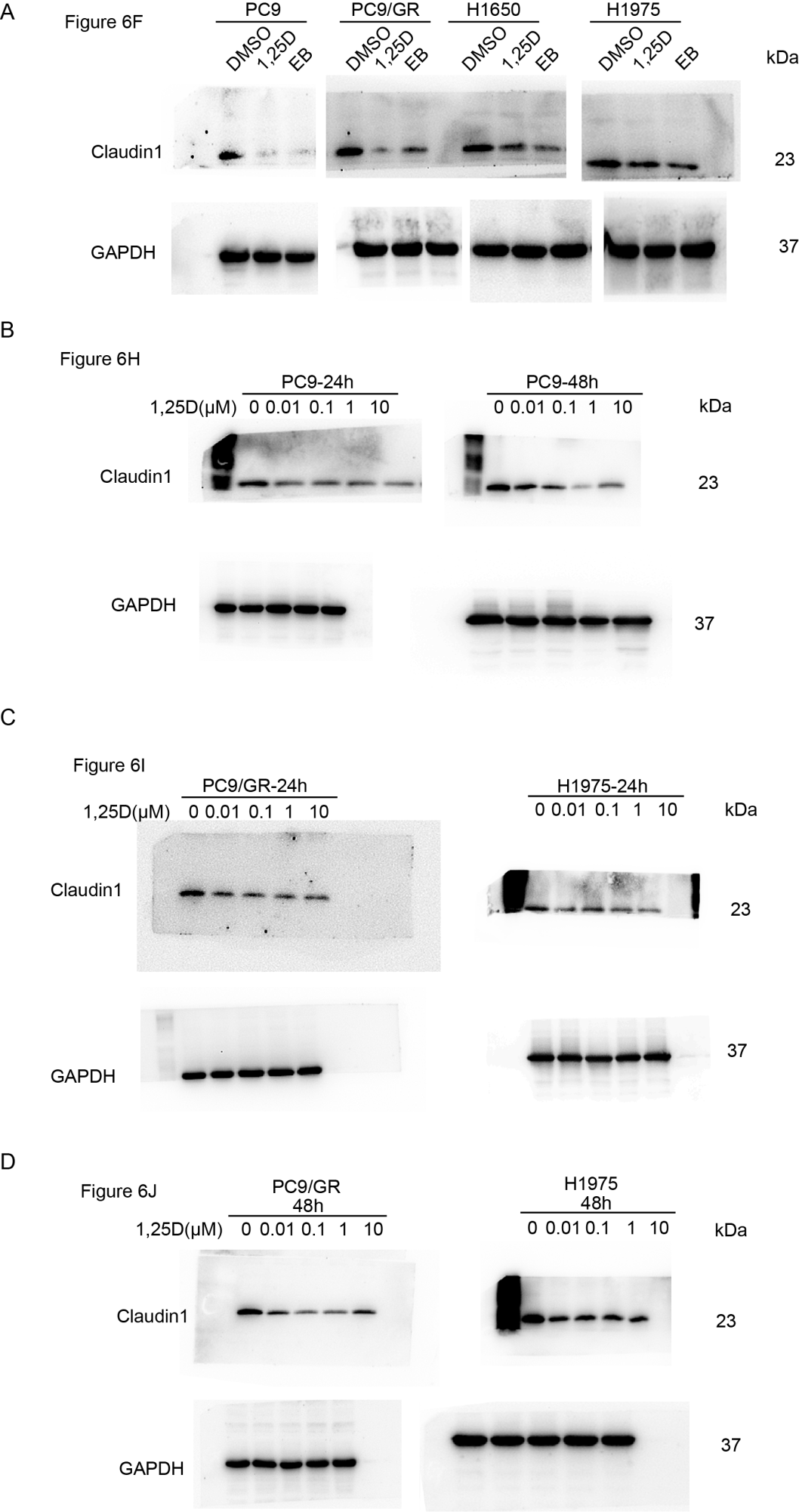


Figure 6-2 Western blot

**Fig. Sg**


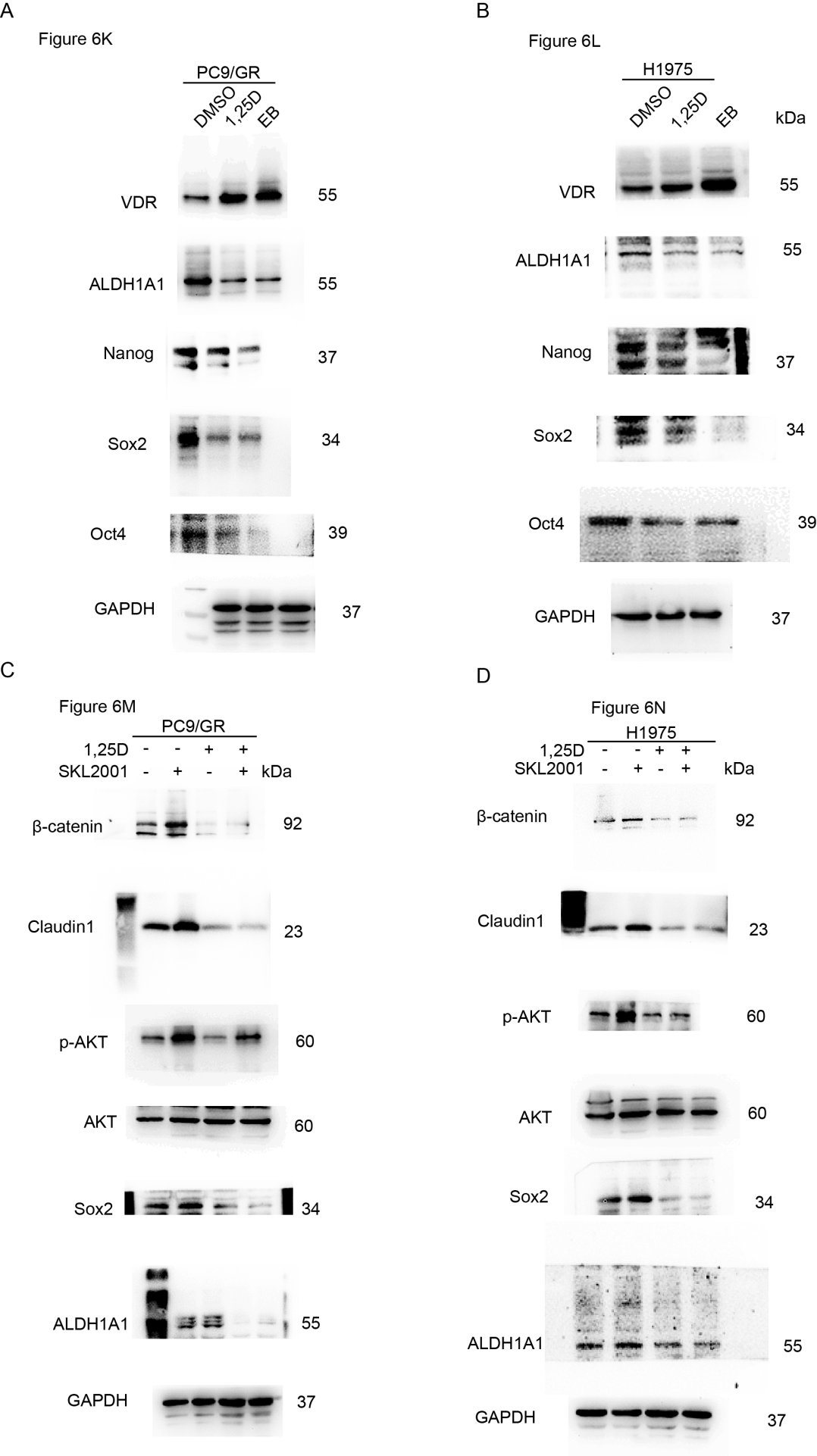


Figure 6-3 Western blot

**Fig. Sh**


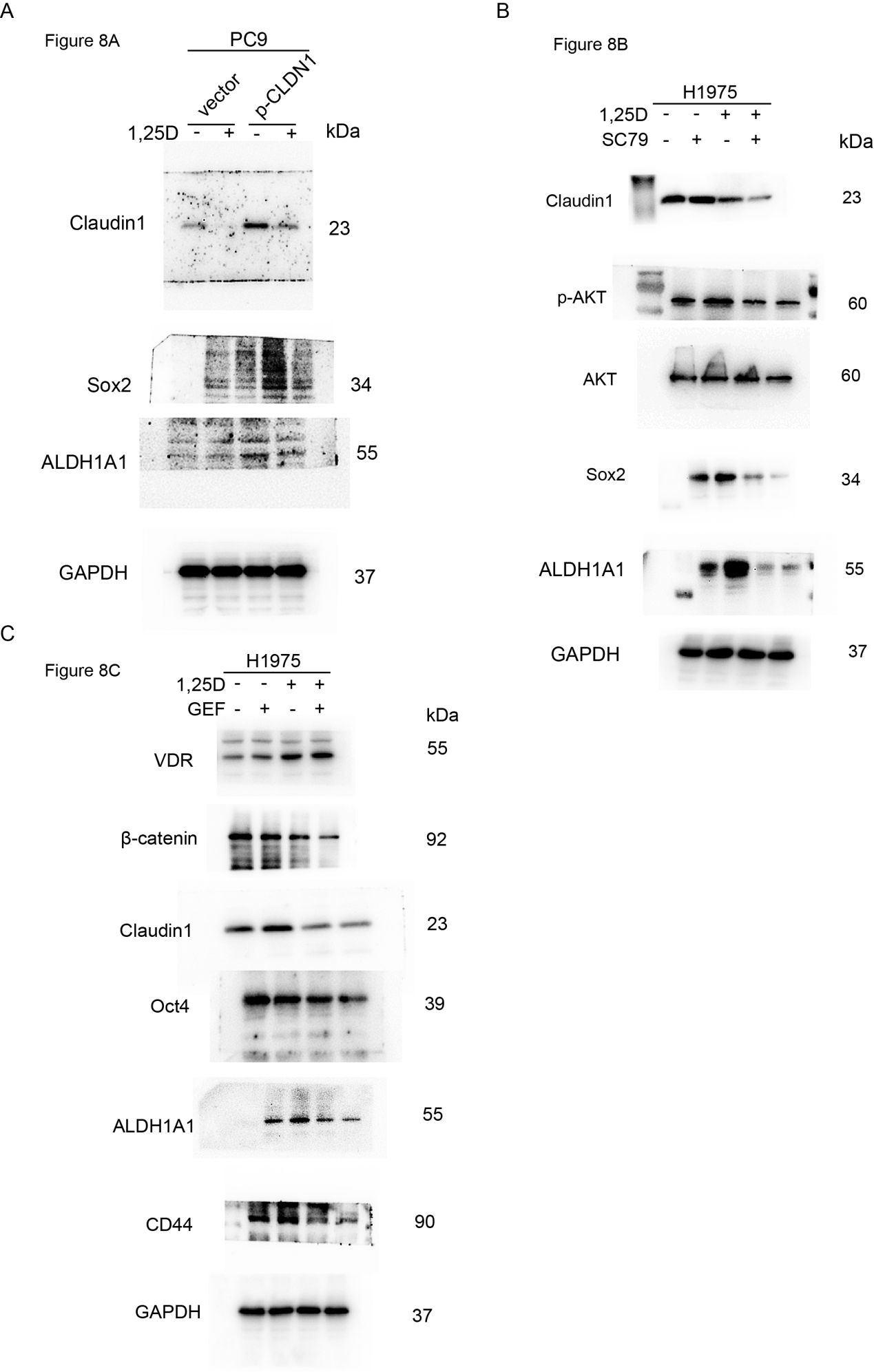


Figure 8 Western blot
