# Supplementary material for: Claudin1 decrease induced by 1,25-dihydroxy-vitamin D3 potentiates gefitinib resistance therapy through inhibiting AKT activation-mediated cancer stem-like properties in NSCLC cells
Source: Cell Death Discov. 2022 Mar 18;8:122. doi: 10.1038/s41420-022-00918-5 (PMC8931006; doi:10.1038/s41420-022-00918-5)
Supplement: Supplementary file 3 — Author contribution form [file 41420_2022_918_MOESM3_ESM.pdf]

DECLARATION OF CONTRIBUTIONS TO ARTICLE

ADMC

Manuscript Number: CDDISCOVERY-21-2025

Journal Name: *Cell Death Discovery* (the 'Journal')

Proposed Title of the Contribution:

Claudin1 decrease induced by 1,25-dihydroxy- vitamin D3 potentiates gefitinib resistance therapy through inhibiting AKT activation-mediated cancer stem-like properties in NSCLC cells (the 'Contribution)

Author(s):

Zhirong Jia, Kaiwei Wang, Yalei Duan, Kaiyong Hu, Yameng Zhang, Meisa Wang, Kang Xiao, Shuo Liu, Zhenzhen Pan, Xuansheng Ding (the 'Authors')

For all *CDDis*covery articles, each person named as an author in the published version must be able to show he or she has contributed substantially to the article.

Authorship credit should be based on 1) substantial contributions to conception and design, acquisition of data, or analysis and interpretation of data; 2) drafting the article or revising it critically for important intellectual content; and 3) final approval of the version to be published. Authors should meet conditions 1, 2 and 3.

Any person who cannot be shown to have made a substantial contribution to the article cannot be listed as an author in the final version. The name of any person who is deemed to have made a minor contribution can, however, appear in the Acknowledgments section of the article.

Please complete the table below to indicate the contributions of all named authors to the manuscript.

Author Full Name:	Specification of Contribution to the Manuscript:
Zhirong Jia and Xuansheng Ding	performed conception and design
Zhirong Jia, Kaiwei Wang and Xuansheng Ding	carried out the development of methodology
Zhirong Jia, Kaiwei Wang, Yalei Duan, Kaiyong Hu, Kang Xiao and Shuo Liu	completed data acquisition
Yalei Duan, Zhirong Jia, Yameng Zhang, Kaiwei Wang, Meisa Wang and Zhenzhen Pan	performed analysis and interpretation of data
Zhirong Jia and Xuansheng Ding	completed the writing, review, and/or revision of the manuscript
Kaiyong Hu and Xuansheng Ding	provided the administrative, technical, or material support
Xuansheng Ding	fulfilled study supervision

Please complete the table below to indicate the contributions of all named authors to the figures.

Figure 1:

Xuansheng Ding and Zhirong Jia designed the experiments; Zhirong Jia completed GEO analysis, cell culture, QRT-PCR and MTT assay; Kaiwei Wang and Yameng Zhang performed sample collecting and western blotting.

Figure 2:

Zhirong Jia completed cell culture,MTT assay and clonal formation assay; Kaiwei Wang and Yalei Duan performed Edu staining and QRT-PCR assay.

Figure 3:

Xuansheng Ding and Zhirong Jia designed the experiments; Kaiyong Hu and Zhenzhen Pan advised on experimental design; Zhirong Jia and Meisa Wang performed lentivirus infection, cell culture and collection; Zhirong Jia, Kaiwei Wang, Yalei Duan, Kaiyong Hu, Meisa Wang, Kang Xiao and Shuo Liu helped the establishment of xenograft tumor and the collection of animal samples. Kaiwei Wang carried out western blotting analysis.

Figure 4:

Zhirong Jia completed cell culture and QRT-PCR assay, Kaiwei Wang performed sample collecting and western blotting analysis. Yameng Zhang carried out the collection of fluorescence images.

Figure 5:

Yalei Duan performed the cell culture and MTT assay, Zhirong Jia performed cell culture, siRNA transfection and drug treatment, Kaiwei Wang and Yalei Duan carried out sample collecting and western blotting analysis.

Figure 6:

Xuansheng Ding and Zhirong Jia designed the experiments; Kaiyong Hu and Zhenzhen Pan advised on experimental design; Zhirong Jia and Yalei Duan completed cell culture, sample collecting, and QRT-PCR analysis; Kaiwei Wang performed western blotting analysis.

Signed for and on behalf of the Author(s):	Print Name:	Date:
Zhirong Jia	Zhirong Jia	07/05/2021
